# Hidden Structural Colors from Bistable, Electrically Driven Covalent Organic Framework Photonic Assemblies for Secure Optical Encoding

**DOI:** 10.1021/acsnano.5c18545

**Published:** 2025-12-18

**Authors:** Tolga Zorlu, Flora Schöfbeck, Julian Lemmel, Daoming Sun, Tanja Eder, Michael R. Reithofer, Jia Min Chin

**Affiliations:** † Department of Functional Materials and Catalysis, 27258 University of Vienna, 1090 Vienna, Austria; ‡ Vienna Doctoral School in Chemistry (DoSChem), 27258 University of Vienna, 1090 Vienna, Austria; § Institute of Inorganic Chemistry, Faculty of Chemistry, 27258 University of Vienna, 1090 Vienna, Austria; ∥ Cyber-Physical-Systems-Group, Faculty of Informatics, Technical University Vienna, 1040 Vienna, Austria; ⊥ Wolfgang Pauli Institute, 1090 Vienna, Austria

**Keywords:** colloidal assemblies, covalent-organic frameworks, optical encoding, photonic materials, stimuli-responsive materials

## Abstract

Optical encryption using nanostructured materials provides a powerful route for secure data encoding. In this work, an electrically reconfigurable colloidal photonic platform based on covalent organic framework (COF) particles is described, enabling dynamic and bistable data encryption. Spatially controlled electrophoretic assembly of monodisperse COF particles within patterned cells produces Bragg reflections that are visible only under bright-field (BF) microscopy as strong broadband scattering from nanoscale particle surface roughness conceals the encoded states from the naked eye. By tuning the synthesis time, the particle surface roughness and, thus, the degree of concealment can be precisely controlled. Unlike conventional optical systems, where scattering degrades visibility, we report it as an intrinsic security feature, transforming a loss mechanism into a tool for optical masking. The demonstrated platform combines electrical addressability, conditional optical visibility, and algorithmic decoding to deliver a compact, multifactor encryption system. These results demonstrate colloidal COF dispersions as a versatile class of photonic materials for secure displays, anticounterfeiting, and adaptive optical communication technologies.

## Graphical abstract

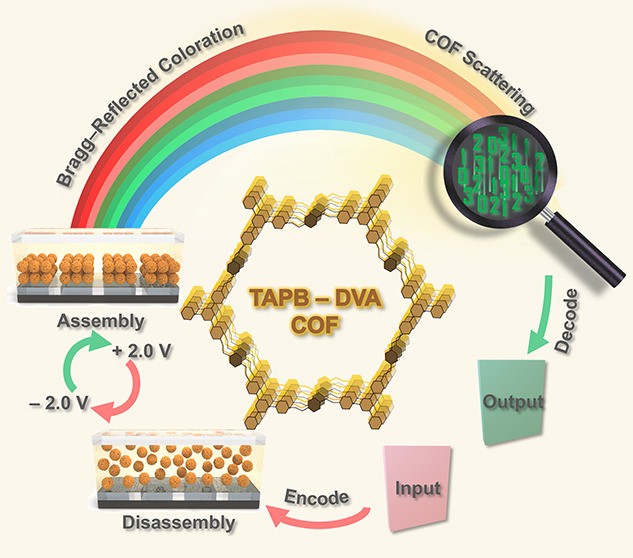

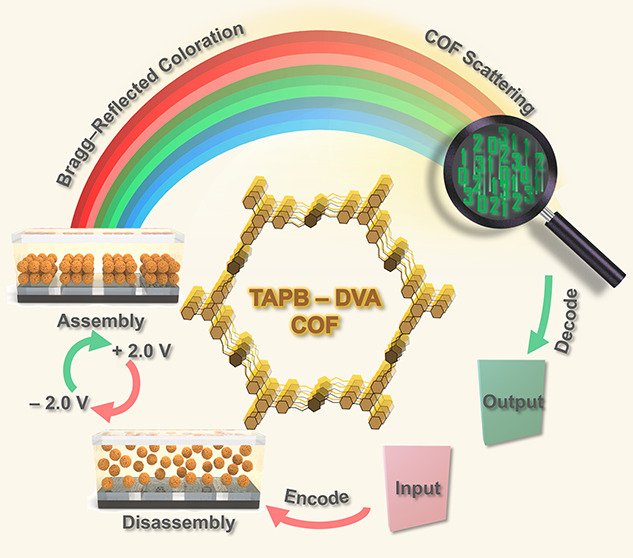

## Introduction

Optical encryption represents a powerful and energy-efficient strategy for secure data storage and communication.^1–3^ Unlike algorithmic cryptography, nanomaterial-based optical systems encode information through intrinsic optical phenomena such as structural coloration,^4,5^ plasmonic resonance,^6,7^ stimulus-responsive behavior,^8–10^ and photonic bandgap engineering.^11,12^ Depending on the optical, physicochemical, and structural characteristics of the selected nanomaterials, these effects can operate singly or in combination, and integrating multiple optical responses within one material would enable multifactor, highly secure encryption, an outcome rarely achievable by using traditional approaches.

A broad spectrum of nanomaterials has been investigated for optical cryptography, including plasmonic metals (e.g., Au and Ag),^13,14^ transition metal dichalcogenides and oxides (e.g., MoS_2_, WS_2_, and graphene oxide),^15–17^ quantum dots,^18^ and polymers.^19^ Recently, porous nanostructures have emerged as particularly attractive for optical applications due to their large surface areas and structural tunability. Among them, covalent–organic frameworks (COFs) stand out for their well-defined molecular architectures, adjustable pore sizes, and modular synthesis, making them ideal for optical platforms. For example, COFs assembled from 1,3,5-tris­(4-aminophenyl)­benzene (TAPB) and benzene-1,3,5-tricarbaldehyde (BTCA) can yield monodisperse colloidal particles that self-organize into photonic crystals (PCs) via controlled solvent evaporation,^20^ exhibiting solvent-responsive structural color. More recently, COFs synthesized from TAPB and 2,5-divinylterephthalaldehyde (DVA) have formed hierarchical photonic architectures through diverse templating approaches,^21,22^ demonstrating their structural versatility. Despite such advances, COFs remain underexplored for optical encryption and secure information storage, as current systems primarily rely on passive modulation via guest-molecule incorporation. Yet, the tunable morphologies and periodic refractive index ranges of COFs offer untapped potential for dynamic, responsive encryption schemes.

Numerous optical encryption platforms have combined multiple responses, for example, spectral or spatial multiplexing, phase-change-based color switching, and metasurface-generated holograms,^23^ and several of these systems already target steganography^11^ or anticounterfeiting applications. However, these typically rely either on naked-eye-visible color or holographic contrast under ambient illumination, or on specialized readout such as coherent lasers, polarization optics,^24,25^ angle- or wavelength-selective detection, or even computational phase reconstruction.^26^ In contrast, the COF-based platform described here combines low-voltage electrical programmability of colloidal photonic assemblies with conditional visibility of the encoded states, where Bragg reflections are invisible in macroscopic view yet can be retrieved under a standard bright-field (BF) microscope and decoded using simple RGB image analysis. Together with bistability and full rewritability, this yields a compact multifactor optical encryption scheme that does not require additional polarization-, wavelength-, or phase-selective optics beyond conventional microscopy.

Here, we present an electrically addressable optical cryptography platform that integrates three security factors based on colloidal COF particles confined within a photonic assembly chip (PAC) (Figure 1a). In this system, COF colloids undergo spatially controlled electrophoretic assembly, producing Bragg-reflected coloration selectively along patterned indium–tin oxide (ITO) electrodes. The nanoscale surface roughness and intrinsic porosity of COFs introduce refractive-index inhomogeneities that amplify light scattering.^27^ While such scattering is typically regarded as an optical loss mechanism that diminishes contrast, here it is harnessed as a built-in security feature, concealing Bragg reflections from direct view, while allowing retrieval only under BF microscopy. This deliberate inversion of a conventional limitation into a functional advantage indicates a scattering-assisted pathway to programmable, high-capacity optical encryption.

**Figure 1:**
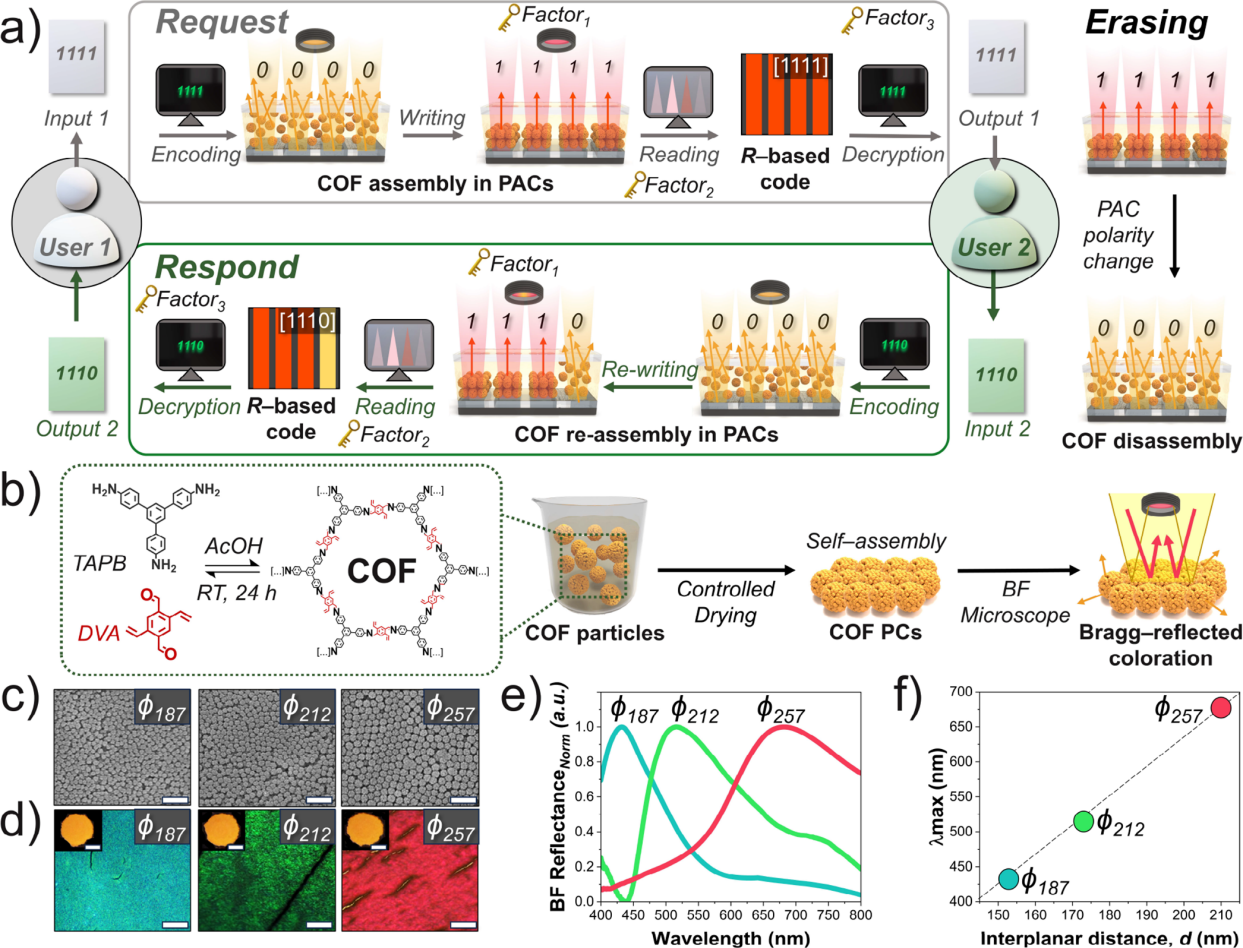
(a) Schematic illustration of the COF-based optical encoding platform. An applied EF (Factor 1) drives electrophoretic assembly of COF colloids along patterned ITO channels, generating Bragg-reflected color (“1”) in biased regions while unassembled areas remain yellow (“0”). The encoded pattern is concealed by the naked eye but visible under BF microscopy (Factor 2) and decoded via an image-processing algorithm (Factor 3). Reversing the field polarity enables a cyclic write–erase–rewrite operation. (b) Preparation of ordered TAPB-DVA photonic superstructures; red and yellow arrows indicate coherent Bragg reflection and incoherent scattering, respectively. (c) FE-SEM images of COF particles. Scale bars: 1 μm. (d) BF optical micrographs of dried photonic superstructures; insets: macrographs. Scale bars: 0.2 mm (main) and 2 mm (insets). (e) Normalized reflectance spectra at θ = 0°. (f) λ_max_ as a function of interplanar spacing *d*_111_.

Once assembled, the photonic structures exhibit a pronounced bistability. The encrypted optical states remain intact for days without power but can be completely erased by reversing the polarity of the applied voltage bias. The resulting patterns are digitally programmable, erasable, and rewritable on demand, providing a direct physical implementation of the binary encryption states. Collectively, these characteristics yield a reconfigurable, low-power optical encryption platform that encodes digital inputs as covert photonic patterns retrievable only under controlled illumination.

## Results and Discussion

### Preparation and Characterization of COF Particles

COF particles with varying sizes were synthesized using DVA and TAPB building blocks in acetonitrile (ACN) at room temperature (28 °C) for 24 h, with acetic acid (AcOH) as the catalyst (Figure 1b). The particle size was simply controlled using different polyvinylpyrrolidone (PVP) concentrations, as confirmed by field-emission scanning electron microscopy (FE-SEM) showing a narrowly dispersed quasi-spherical morphology (Figures 1c and S1–S3). The particles were measured to have diameters, *ϕ*, of 187 ± 13, 212 ± 8, and 257 ± 8 nm and are denoted as *ϕ*_187_, *ϕ*_212_, and *ϕ*_257_, respectively (Figure S4). As previously reported, PVP tunes COF particle size by adsorbing onto growing crystal surfaces, inhibiting growth and yielding smaller, uniform particles.^21^ Raman spectroscopy (λ_excitation_ = 785 nm) shows the vibrational properties of the COF particles (Figure S5). The COF Raman spectrum lacked the C=O stretch of DVA (1681 cm^–1^) and NH_2_ wagging of TAPB (1354 cm^–1^) but showed a distinct C=N stretch at 1584 cm^–1^, confirming imine linkage formation (Figure S6).^28^ Powder X-ray diffraction (PXRD) patterns also confirmed the crystalline structure, aligning with reported imine-linked COF models.^29–31^ A characteristic peak at 2.73° corresponds to the (*100*) plane, with additional reflections at 4.75°, 5.51°, 7.34°, and 9.68° assigned to the (*110*), (*200*), (*210*), and (*220*) planes, respectively (Figure S7), and the patterns closely match eclipsed AA stacking models.^32–34^ N_2_ physisorption analysis at 77 K shows a type IV isotherm, typical of mesoporous materials, with Brunauer–Emmett–Teller (BET) surface areas (*S*_BET_) of 1441, 1376, and 1556 m^2^ g^–1^ for *ϕ*_187_, *ϕ*_212_, and *ϕ*_257_, respectively (Figures S8–S10). Density functional theory (DFT) pore size analysis also indicates a dominant pore size of 3.53 nm with minor contributions from 2.58 nm pores (Figures S11–S13).

### Photonic Response

To characterize the photonic response of as-prepared COF particles, we washed and redispersed them in ACN (25 mg mL^–1^) and deposited 50 μL of droplets from these dispersions onto clean glass surfaces. The droplets were dried at 120 °C for 5 min for assembly into photonic superstructures. The particles formed a face-centered cubic (*fcc*) lattice, with (*111*) and (*11̅1*) planes consistent with prior reports (Figure S14).^35^ The photonic superstructures appear yellow by eye due to incoherent light scattering (Figure 1d, inset photos), while their structural coloration is distinctly observable via BF optical microscopy (Figure 1d), and the reflectance spectra at normal incidence to a flat sample (defined as θ = 0°) revealed Bragg reflection maxima (λ_max_) at 432, 515, and 664 nm for *ϕ*_187_, *ϕ*_212_, and *ϕ*_257_, respectively (Figure 1e). These λ_max_ values align with the interplanar distances (*d*) of the (*111*) planes (*d*_111_) and the Bragg–Snell law at normal incidence (eq 1),^36^ as plotted in Figure 1f:

$$\lambda_{\max}=2\times n_{\mathrm{eff}}\times d_{111}$$ 1

Here, *d*_111_, the interplanar distances in the COF assemblies are given by 0.816 × *ϕ*,^37^ and are calculated to be 153, 173, and 210 nm for *ϕ*_187_, *ϕ*_212_, and *ϕ*_257_, respectively. Applying the Bragg–Snell law (eq 1) yielded effective refractive indices (*n*_eff_) of 1.41, 1.47, and 1.58 for dry photonic assemblies of *ϕ*_187_, *ϕ*_212_, and *ϕ*_257_, respectively. The refractive indices of the COF particles (*n*_COF_) were then calculated using the effective dielectric (ε) function by using eq 2:

$$\varepsilon_{\mathrm{eff}}={n_{\mathrm{eff}}}^{2}=({n_{\mathrm{COF}}}^{2}\times \varphi_{\mathrm{COF}})+({n_{\mathrm{medium}}}^{2}\times \varphi_{\mathrm{medium}})$$ 2

Here, φ_COF_ (0.74) and φ_medium_ (0.26) are the volume fractions of COFs in air (*n*_medium_ = 1), respectively, assuming perfect spherical particles in the *fcc* (*111*) lattice, and omitting particle porosity.^38^ *n*_COF_ values were calculated as 1.53, 1.60, and 1.74 for *ϕ*_187_, *ϕ*_212_, and *ϕ*_257_, respectively, and these values match reported data for other COF structures.^20,39^ Chromaticity coordinates from the reflection spectra at normal incidence, plotted on the CIE-1931 diagram (Figure S15), corroborate visual observations of the superstructures’ color.

### Preparation of Colloidal COF Dispersions

COF particles of varying sizes were dispersed in propylene carbonate (PCb, 25 mg mL^–1^) to obtain colloidal dispersions for testing their electrophoretic behavior. PCb was selected for its low volatility, chemical stability, and high dielectric constant (ε_PCb_ = 64), which promotes efficient electrophoretic motion of charged particles under an electric field (EF).^40–42^ However, unmodified COF particles rapidly aggregate in PCb and lose their colloidal stability (Figure S16), as indicated by high polydispersity indices (PDI ≈ 0.9–1.0) and enlarged hydrodynamic diameters measured by dynamic light scattering (DLS) (Figure S17). To overcome this, we added cetyltrimethylammonium bromide (CTAB) to COF dispersions in PCb (COF/PCb), functionalizing the particles to obtain stable and homogeneous colloids. CTAB increased the interparticle electrostatic repulsion, shifting the zeta potential, ζ, of COF particles to +25, +27, and +33 mV for *ϕ*_187_, *ϕ*_212_, and *ϕ*_257_, respectively, compared to nonfunctionalized samples (−5, −4.5, and −2 mV, respectively) (Figure S18). This kept the particles dispersed in solution without sedimentation for an extended period (Figure S19), showing low PDI values (≈0.17–0.19) and retained stability over at least 24 h (Figure S20).

### Electrophoretic Assembly Profiles of COF Colloids

Following postsynthetic modification, the COF/PCb dispersions were loaded into a cell comprising two unpatterned ITO-coated glass slides, separated by an adhesive spacer and connected to a direct-current (DC) power supply (Figures 2a and S21). Upon applying a voltage bias (−2.0 V), the COF particles underwent electrophoretic assembly, giving rise to Bragg reflection-driven photonic coloration.

**Figure 2:**
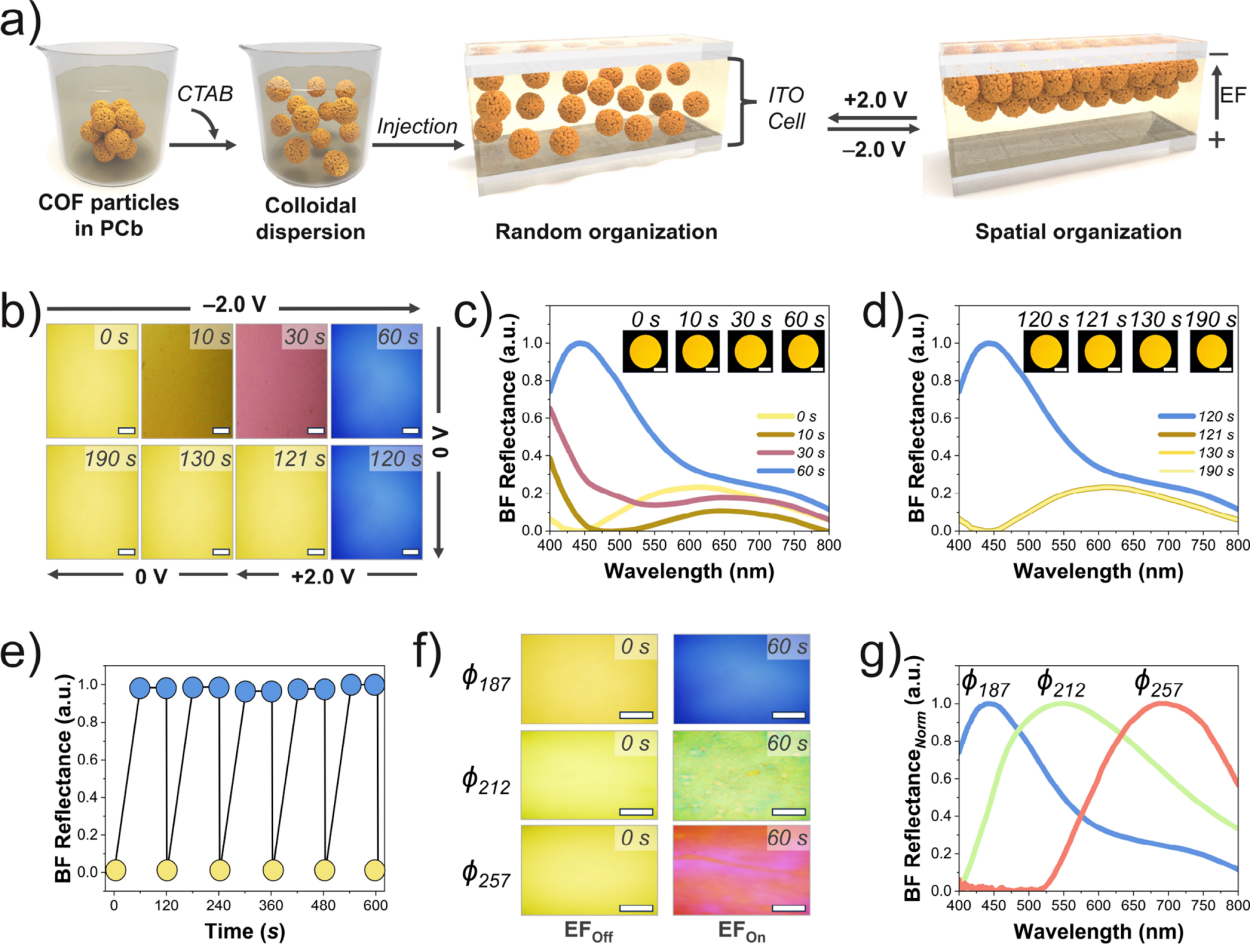
(a) Schematic of CTAB functionalization and COF electrophoretic assembly. (b) Time-dependent BF optical micrographs showing structural color changes in COF/PCb dispersion (*ϕ*_187_) at θ = 0° under EF: −2.0 V applied from *t* = 0–60 s, followed by +2.0 V from *t* = 120–121 s. Scale bars: 0.2 mm. (c and d) Corresponding reflection spectra at θ = 0° from 0–60 s and 120–190 s, respectively. Insets: macrographs at different times. Scale bars: 2 mm. (e) Cyclic performance of COF/PCb (*ϕ*_187_) under different applied voltage biases (blue = −2.0 V and yellow = +2.0 V). (f) Structural colors of COF/PCb electrophoretic assemblies with different sizes at θ = 0°. Scale bars: 0.2 mm. (g) Normalized reflectance spectra of COF/PCb electrophoretic assemblies with different sizes after 60 s at θ = 0°.

The EF-induced optical response was monitored by BF optical microscopy and direct observation. Initially, the COF/PCb dispersion (*ϕ*_187_ as a model) appears yellow under a BF microscope, in the absence of an applied voltage (EF_Off_) (Figure 2b, 0 s). Applying a voltage bias of −2.0 V (EF_On_) triggers particle accumulation and partial ordering at the cathode, producing a red reflection that gradually evolved to blue within 60 s (Figure 2b). The optical transition can be followed via BF microscopy but appears visually unchanged by eye (inset photos, Figure 2c). The corresponding reflectance spectra at θ = 0° confirm the color evolution over time (Figure 2c).

The transient spectral changes reflect the different assembly stages. During the early stage (0–10 s), positively charged CTAB-functionalized COF particles (ζ = +25 mV) migrate and form a loosely packed assembly with a large interplanar spacing (*d*_1__1__1_) and high effective refractive index (*n*_eff_), producing a red-shifted, broadened reflection. As electrophoretic deposition proceeds (10–60 s), progressive compaction decreases *d*_1__1__1_, strengthens long-range order, and shifts the Bragg peak toward shorter wavelengths (λ_m__a__*x*_ ≈ 442 nm), consistent with denser *fcc* packing. This red-to-blue spectral evolution parallels observations in the field- or gravity-induced assembly of colloidal photonic crystals.

When the EF is switched off (0 V), the structural color persists for at least 2 days (Figure 2b, 120 s, and S22), demonstrating bistable assembly. A comparable phenomenon has been observed with SiO_2_ particles, where electrophoretic deposition resulted in the formation of a stable, colorless aggregate that persisted even after the external EF was removed, attributed to the locally increased viscosity at high particle volume fractions. Such bistability is advantageous for optical encryption and display technologies because it maintains the encoded state without continuous power input.

Reversing the polarity (+2.0 V) rapidly redisperses the COF particles (<1 s) through electrostatic repulsion, fully restoring the initial yellow state (Figure 2b, 121 s, and S23). Reflectance spectra after polarity reversal (Figure 2d) confirm that the color change is completely reversible over multiple cycles (Figure 2e).

Similar EF-driven behavior was observed for *ϕ*_212_ and *ϕ*_257_ nm COFs (Figure 2f). Reflectance spectra recorded after 60 s (Figure 2g) show λ_ma*x*_ values of 442, 547, and 690 nm for *ϕ*_187_, *ϕ*_212_, and *ϕ*_257_ dispersions, respectively. Relative to dry assemblies, these reflections are red-shifted due to higher *n*_eff_ arising from the replacement of air in the interparticle voids and COF pores by PCb (*n*_PCb_ = 1.421),^43^ consistent with prior reports of red-shifts in porous photonic materials caused by solvent infiltration.^20,44^ Using the Bragg–Snell law in eq 1, and assuming a similar *d*_111_ as in the dry assemblies, we calculated *n*_eff_ for the COF assemblies in PCb as 1.44, 1.58, and 1.64 for ϕ_187_, ϕ_212_, and ϕ_257_, respectively. The chromatic evolution in CIE-1931 space (Figure S24) further illustrates this red-shift.

To optimize color contrast, dispersions of different particle concentrations (1.0–2.5 wt %) were assembled at −2.0 V for 60 s (Figure S25). A concentration of 2.5 wt % yielded the highest reflectance (Figure S26) while retaining colloidal stability; higher concentrations caused aggregation. Voltage optimization revealed −2.0 V as the minimal bias, achieving complete ordering, as stronger fields (−2.5 to −4.0 V, Figures S27 and S28) produced no additional spectral shift or intensity increase, indicating saturation of electrophoretic packing. Thus, −2.0 V represents the optimized operating point for generating reversible, well-defined photonic responses in these COF systems.

### Role of Surface Roughness in Bragg-Reflected Coloration

To elucidate the optical origin of the COF-based structural coloration, we examined how nanoscale surface texture influences Bragg reflection in superstructures of dry COF samples and COF/PCb colloids under applied fields. The observed coloration arises from an interplay between coherent Bragg reflection and strong incoherent broadband scattering induced by surface roughness and local refractive-index variations within the quasi-spherical COF particles.^45,46^ This scattering component dominates the optical response, masking angle-dependent Bragg reflections and thereby governing the overall visibility of the photonic color.

We prepared a series of COF samples based on the synthesis protocol for *ϕ*_257_, varying the reaction time to systematically tune the particle roughness. Rapid nucleation occurred within 5 min (Figure 3a), and both particle size and roughness increased with reaction time up to 24 h (Figures S29–S32). FE-SEM images revealed particle sizes of 213 ± 13, 217 ± 11, 225 ± 10, and 234 ± 12 nm, respectively (Figures 3b and S33), reaching the target size of 257 ± 8 nm at 24 h. Extending the synthesis to 72 h did not further increase particle size (Figure S34) but progressively transformed the particles from relatively smooth spheres into rough, urchin-like morphologies, in agreement with previous reports.^47,48^ The reversible nature of COF imine linkages enables defect repair and surface recrystallization, promoting crystallite growth and increased roughness that enhance incoherent scattering over a wide angular range and reduce apparent color intensity without changing the photonic origin of reflection (Figure 3c).^49–52^ The influence of roughness is also macroscopically evident. The COF superstructure obtained by drying the sample after 5 min of reaction (*ϕ* = 213 nm, smooth particles) exhibited a vivid green reflection visible to the naked eye and under BF microscopy (Figures 3d and S35a), whereas an ordered superstructure from similarly sized but rougher *ϕ*_212_ particles (24 h synthesis) showed no visible structural coloration (Figures 3e and S35b).

**Figure 3:**
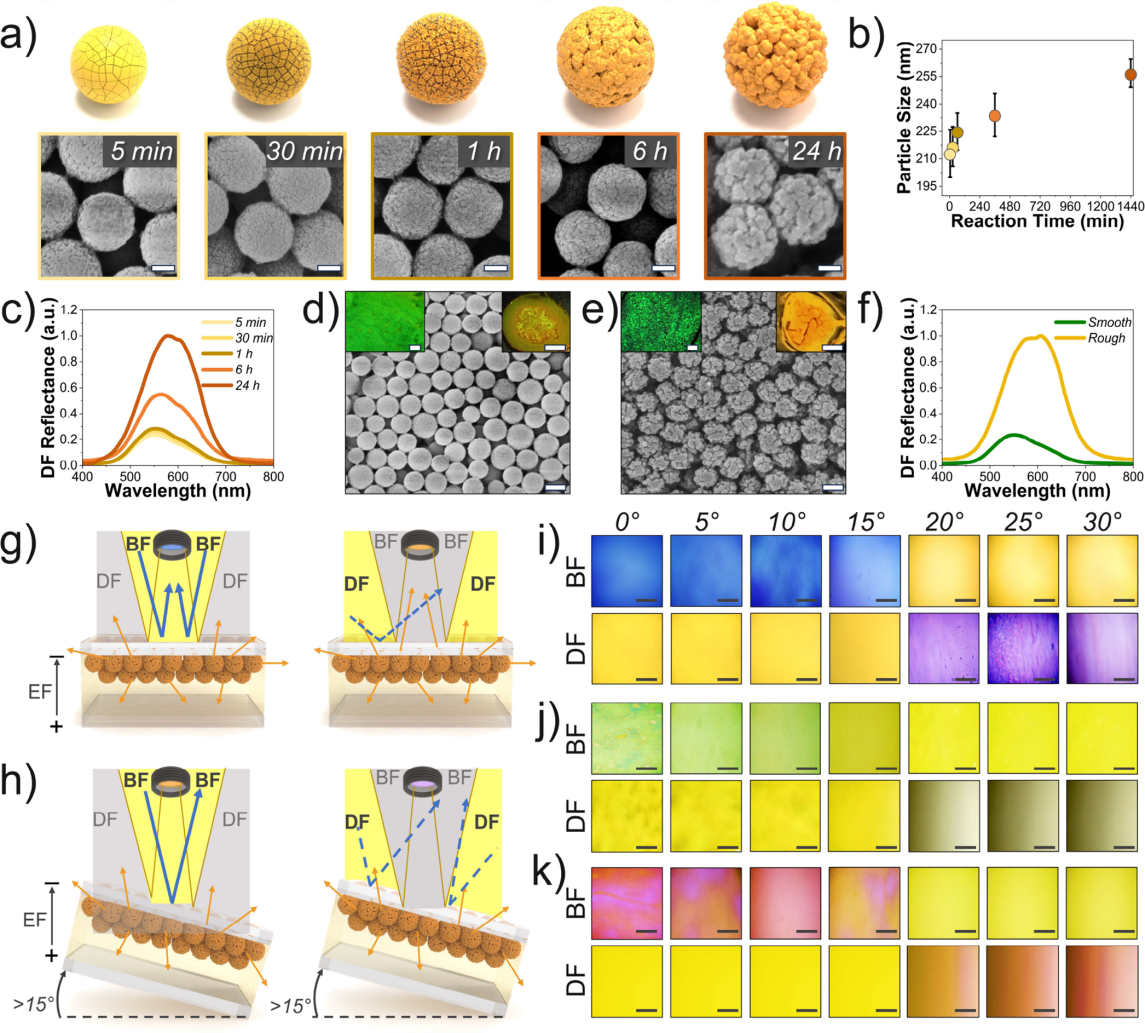
(a) FE-SEM images showing surface evolution of the COF particles (257 ± 8 nm) with reaction time. Scale bars: 50 nm. (b) Particle diameter vs reaction time. (c) Corresponding DF scattering spectra at normal incidence. (d,e) FE-SEM images of dried *ϕ*_213_ (smooth) and *ϕ*_212_ (rough) superstructures; scale bars: 200 nm; insets: BF micrographs (upper left) and macrographs (upper right). Scale bars: 0.2 mm and 2 mm for BF micrographs and macrographs, respectively. (f) Corresponding DF scattering spectra of the dried photonic superstructures. (g,h) Schematic of BF and DF microscope illumination geometry for untilted samples and tilted samples (θ > 15°). (i–k) Optical micrographs of the COF/PCb dispersions after EF application at varying incidence angles under BF and DF. Scale bars: 0.2 mm.

Angle-resolved reflectance spectra of the dried smooth (*ϕ* = 213 nm) and rough *ϕ*_212_ assemblies measured in a colinear geometry (0–30°, 5° steps; Figure S36) show distinct optical behaviors. The rough sample shows a single broad maximum that red-shifts with an increasing tilt. This apparent red-shift arises because a longer optical path at oblique incidence enhances attenuation of short wavelengths, which are scattered and absorbed more strongly. In contrast, the smooth sample exhibits two features: a Bragg band at ∼500 nm and a broad long-wavelength peak (∼750 nm), separated by a dip (550–680 nm) where diffuse scattering is minimal. Under tilt, the short-wavelength feature shows only an overall slight red-shift due to the competing effects of path-length-induced red-shifting and the intrinsic Bragg–Snell blue-shift of the (*111*) reflection. The long-wavelength band is attributed to thin-film interference (Fabry–Pérot) within the assembled colloidal film, following *m*λ = 2*n*_eff_ *t*cos θ,^53^ and therefore blue-shifts with increasing tilt. Its absence in the rough assemblies confirms that surface irregularity and strong diffuse scattering suppress coherent interference across the film thickness. Dark-field (DF) scattering spectra, recorded with a fiber-coupled spectrometer integrated with the microscope,^54^ showed significantly stronger diffuse scattering of the rough vs smooth samples (Figure 3f). Additionally, DF scattering spectroscopy of dried *ϕ*_187_, *ϕ*_212_, and *ϕ*_257_ assemblies showed that increasing particle sizes produces a pronounced increase in scattering intensity (Figure S37).

Having established the effect of surface roughness and particle size on the optical behavior of dried assemblies, we next examined the electrophoretically assembled COF/PCb dispersions under the applied fields. Despite the surface roughness of *ϕ*_187_, *ϕ*_212_, and *ϕ*_257_, BF reflection microscopy selectively collects coherently reflected light (Figure 3g,h), revealing the intrinsic Bragg colors of their electrophoretic assemblies. Accordingly, the *ϕ*_187_, *ϕ*_212_, and *ϕ*_257_ assemblies appeared distinctly blue, green, and red. Tilting the COF/PCb cell confirmed the interplay of Bragg reflection and scattering. At small angles (θ ≤ 15°), specular Bragg reflections dominate, producing vivid coloration (Figure 3i–k, top). Beyond 15°, the reflections exit the optical collection cone, and diffuse scattering prevails, causing the apparent color to move toward yellow. In contrast, DF optical microscopy, which collects oblique scattered light, rendered all samples yellow (bottom rows), and scattering remains strong at all angles, while weak Bragg bands re-emerge above 15°. Corresponding reflectance spectra show decreasing intensity and chromaticity clustering in the yellow region with increasing tilt (Figures S38 and S39) as decreased Bragg-reflected light reaches the detector and diffuse scattering prevails.

### Optical Encoding Concept via Electrophoretic COF Assembly

To evaluate the potential of COF/PCb dispersions for optical encryption, colloidal COF/PCb particles (*ϕ*_257_, as a model) were injected into a cell composed of an unpatterned ITO cover glass and a bottom interdigitated electrode (IDE) substrate, forming a photonic assembly chip or PAC. Each line electrode represented a binary digit, where electrode regions without COF assembly were assigned “0”, those exhibiting particle ordering were assigned “1”. The pattern evolution under an applied EF was monitored by BF microscopy.

At the initial state (Figure 4a­(i)), without an applied voltage, the COF particles remained randomly dispersed and appeared as three yellow lines corresponding to [000] (Figure 4b­(i)). Applying a −2.0 V bias for 60 s to all line electrodes induced electrophoretic assembly along them, generating red Bragg reflections and switching the pattern to [111] (Figure 4a­(ii),b­(ii)). Reversing the polarity dispersed the particles within 1 s, restoring the [000] state, and reapplying a 2.0 V bias to different line electrodes enabled new combinations such as [101] or [010] (Figure 4a,b­(iii,vi)). The regions between electrodes appeared black because the light-absorbing substrate suppressed the background reflection and enhanced the color contrast. These results confirm the spatial selectivity and full reversibility of the electrophoretic writing process. Importantly, the encoded lines remained invisible to the naked eye (Figure 4c).

**Figure 4:**
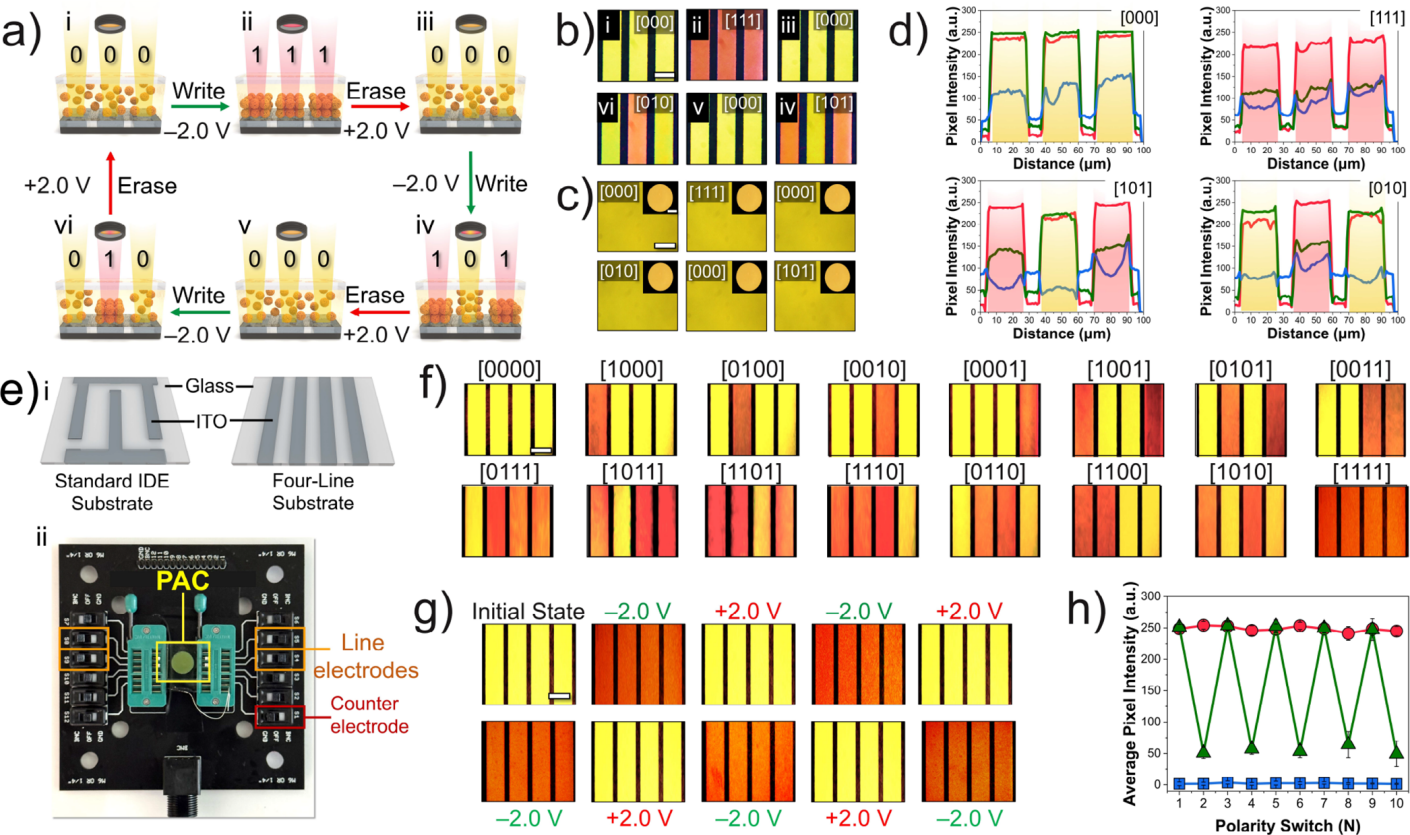
(a) Schematic of the EF-induced write/erase/rewrite cycle in the cell. (b) BF optical micrographs of *ϕ*_257_ superstructures after each cycle; codes are shown in the upper right corner. Scale bars: 25 μm. (c) DF optical micrographs of *ϕ*_257_ superstructures after each cycle; insets: macrographs. Scale bars: 25 μm (main) and 2 mm (insets). (d) Pixel intensity–distance plots from line scans in (b). (e) i. Schematic of standard IDE substrate and four independently line-channeled substrate; ii. photograph of a zero-force insertion board bearing a 4-line PAC. (f) BF optical micrographs of the 16 possible line patterns using the four independent line-channel substrate. Scale bars: 100 μm. (g) BF optical micrographs of *ϕ*_257_ superstructures cycling between the [0000] and [1111] states. Scale bars: 100 μm. (h) Average pixel intensity and polarity switch number relationship of *ϕ*_257_, corresponding to states in (g).

To quantitatively verify the encoded states, we analyzed pixel intensities from the BF micrographs (details in the SI Python scripts). The extracted RGB profiles for different patterns ([000], [111], [101], and [010]) corresponded closely to the optical observations (Figure 4d). Lines containing ordered COF superstructures showed enhanced red intensity from the *ϕ*_257_ assemblies, whereas disordered lines exhibited balanced red–green components, consistent with their yellow appearance. These profiles enabled the reliable digital decoding of the optically written information.

Although each electrode channel operates in a binary ON/OFF manner, the color of the ON state can be tuned by selecting a COF assembly of different particle sizes. PACs filled with *ϕ*_187_, *ϕ*_212_, and *ϕ*_257_ COF/PCb dispersions produced blue, green, and red reflections, respectively, assigned values “3”, “2”, and “1”, respectively, and representative binary patterns, such as [303] and [222], could be successfully written (Figure S40). Although the numerical coding within each cell remains binary, the use of distinct colors introduces a wavelength-selective dimension that could enable spectral multiplexing and concealed information layers. This color tunability therefore provides an additional optical key for multifactor encryption, rather than an increase in bit depth.

While the IDE configuration enabled proof-of-concept encoding, its geometry limited addressable combinations to four, as the first- and third-line electrodes were electrically interconnected (Figure 4e, top). In principle, “*n*” independent lines can generate “2^*n*^” distinct combinations. To overcome this limitation, we fabricated a four-channel ITO substrate through localized etching of the interdigitated electrode to yield four fully independent electrodes (Figure 4e,i, right), increasing the possible states from four to 16 (2^4^) (Figure 4f). As the complexity of the PAC architecture and the number of independent electrodes increased, reliable electrical interfacing became essential. Accordingly, a zero-insertion-force (ZIF) test board was employed to connect the four-line electrodes, enabling stable and reproducible addressing of the four-line PAC (Figure 4e­(ii)). Using *ϕ*_257_ dispersions, distinct 4-bit binary patterns were reproducibly written, erased, and rewritten (Figures S41 and S42).

### Stability and Reproducibility of PACs

To assess the stability, reproducibility, and readout fidelity of the photonic assemblies, the [1111] code was selected as a representative example. The Bragg-reflected coloration consistently exhibited the characteristic red hue of the *ϕ*_257_ assemblies after each polarity-switching cycle (Figure 4g) while RGB-intensity analyses verified consistent digital outputs across repeated cycles (Figure S43). Minor spatial hue variations between individual strips originated from local differences in electrode geometry, film thickness, or COF packing density rather than from temporal instability of the encoded stated.

Quantitative color analysis confirmed a good optical stability. The color-intensity profiles recorded after each cycle (Figure S43) and the average pixel intensities plotted vs switch number (Figure 4h) showed that each strip maintained a constant optical response throughout repeated cycling. The red-channel intensity remained effectively constant, while the green-channel signal periodically aligned with the red signal for the [0000] state and decreased by approximately 5-fold for the [1111] state. The blue-channel intensity remained negligible across all tests, confirming the spectral purity of the reflection. Repeated polarity switching demonstrated excellent temporal stability and reproducibility of the Bragg reflection under applied EF. Furthermore, when the encoded patterns were processed using the programmed line reader, the extracted digital codes precisely matched the intended input sequences, verifying that the optical encoding and pixel reading processes were fully consistent and reliable across multiple write/erase cycles.

### Design of Multi-PAC Arrays for Enhanced Security and High-Capacity Optical Cryptography

Building on the 16 distinct 4-bit code combinations demonstrated above, we extended the concept to multi-PAC arrays, where the sequential readout of multiple PACs and the increased number of independent bits provide higher encoding capacity and security. The model array comprises six independently addressable PACs (6-PAC), each hosting a single COF sample, arranged in two rows (A and B) and three columns (1–3) (Figure 5a). This configuration, with a total of 24-line electrodes, equivalent to 24 bits or 3 bytes, increases the total number of possible combinations exponentially, yielding 2^24^ distinct code permutations and substantially expanding the addressable encoding space compared to a single PAC.

**Figure 5:**
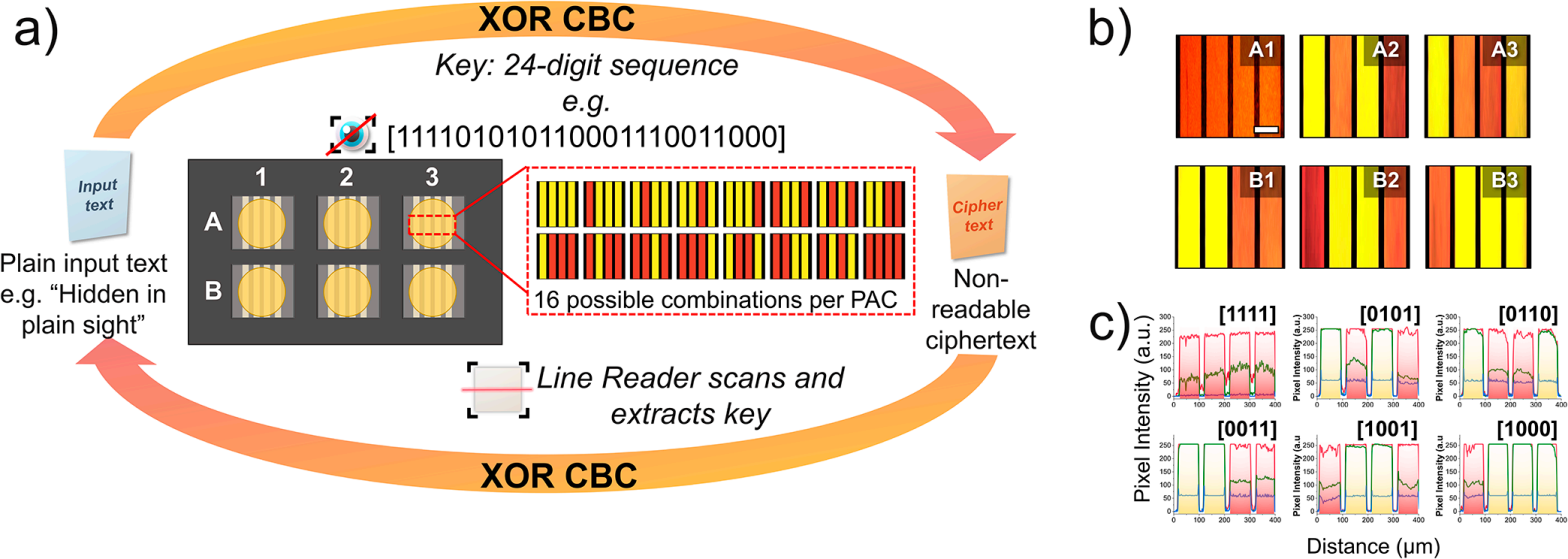
(a) Schematic illustration of the XOR cipher block chaining (CBC) encryption and decryption method. The 24-digit encryption key is depicted by the 6-PAC array and extracted via a customized line reader. (b) BF optical micrographs of *ϕ*_257_ superstructures corresponding to the selected 24 bit binary code. Scale bars: 100 μm. (c) Pixel intensity–distance plots from line scans corresponding to the selected 24 bit binary code.

As a proof-of-concept, we demonstrated the use of the 6-PAC array for encoding digital sequences that serve as encryption keys in an information-encryption workflow (Figure 5a). A simplified exclusive-OR (XOR) cipher combined with a block-chaining (CBC) mechanism was implemented, in which each character of the input message is processed bit-by-bit with both the previously encrypted character and a byte from a binary encryption key. Applying the XOR operation converts readable text into ciphertext, while the same operation applied in reverse recovers the original message. While the XOR cipher is implemented here as a simplified digital simulation, it exemplifies how optically generated binary outputs from the 6-PAC array could be coupled to support cryptographic algorithms for secure data processing.

For demonstration, the 24 bit encryption key [111101010110001110011000] was arbitrarily defined. Voltage-biased activation of selected electrodes within the 6-PAC array produced the expected line patterns visible under BF microscopy (Figure 5b). Sequential readout of the array (A1 → A2 → A3 → B1 → B2 → B3) by a custom line-reader algorithm extracted and converted RGB profiles (Figure 5c) into a digital sequence, precisely reproducing the original input by yielding an output identical with the defined encryption key [111101010110001110011000] (see Python scripts and instructions in the Supporting Information). Correct key retrieval subsequently enabled execution of the XOR cipher, converting the plaintext message *“Hidden in Plain Sight”* into ciphertext and, upon reverse processing, restoring the original text. Polarity reversal erased the encoded information, as evidenced by the disappearance of the red color under BF microscopy and the corresponding RGB intensity profiles (Figure S44).

To regenerate a new encryption key, the field polarity is reversed, restoring the ITO channels to their initial yellow state. Once erased, a new message, for example, [100101101111010101111101], can be written by repeating the encoding–decoding cycle in the opposite direction. The assemblies remain bistable for up to 2 days after field removal, enabling temporary data storage and portable encrypted communication.

## Conclusions

We report an electrically reconfigurable colloidal photonic platform based on TAPB–DVA COF particles that enables cyclic, bistable, and optically concealed data encoding. Through the electrophoretic self-assembly of size-tuned COF colloids within patterned PACs, numerical information is dynamically written as spatially resolved Bragg reflections only visible under BF microscopy. Intrinsic nanoscale surface roughness generates strong scattering, which is transformed from a conventional optical loss mechanism into an integrated security feature for optical masking.

The platform integrates electrical addressability, microscopy-dependent visibility, and algorithmic decoding within a single rewritable device. A negative bias induces rapid field-assisted COF assembly, whereas polarity reversal achieves instantaneous erasure and full reusability without degradation. The encoded states remain stable for days after field removal, reflecting the intrinsic bistability of the assembled COF lattices and eliminating the need for continuous energy input.

Scaling to multi-PAC arrays exponentially expands the addressable coding space and enables wavelength-selective, high-capacity optical encryption. This strategy demonstrates the use of COFs as a platform for programmable photonic logic, anticounterfeiting, and adaptive display technologies, bridging molecular design with reconfigurable device-level functionality.

## Methods

### Chemicals

DVA and TAPB were purchased from TCI Deutschland GmbH. PVP (25K) was purchased from Carl Roth GmbH & Co. PCb, AcOH, isopropyl alcohol, acetone, and ACN were purchased from Merck KGaA. CTAB was purchased from Alfa Aesar Co. ITO-coated glass slides (1.10 mm thick, of which ITO thickness is 100 nm, 20 Ω, both line-patterned and unpatterned ones) were purchased from Ossila Ltd. Double-sided spacers (SecureSeal Imaging Spacers, 9 mm ID × 0.12 mm depth) were purchased from Grace Bio-Laboratories Inc. Insulating tape (HUP tape-15) was purchased from Haupa GmbH & Co. All reactants were used without further purification. Milli-Q water (18 MΩ cm^–1^) was used in all of the aqueous solutions.

### Material Synthesis

COF particles with different sizes were prepared according to the previous report with some modifications.^21^ Briefly, 2.5 mL of TAPB (0.032 M) in ACN was mixed with 2.5 mL of DVA (0.048 M) in ACN via vortex for 10 s. Then, the mixture was filtered with a 0.22 μm filter to obtain a clean yellowish mixture. After this, 1 mL of AcOH (17.5 M) was injected into the mixture and vortexed for 10 s, and it was left undisturbed at room temperature for 24 h. To prepare COFs of three different sizes, before AcOH injection, we added 0, 100, and 200 μL PVP solution in ACN (2 mg mL^–1^), respectively, and left them it undisturbed at room temperature for 24 h. The resulting particles were centrifuged at 6000 rpm for 30 min, washed three times with ACN and three times with acetone, and dried under N_2_. The sizes of COFs were measured using software (ImageJ version 1.54j) based on FE-SEM images. 100 particles were counted in each measurement.

### Self-Assembly of COF Particles

50 μL of each as-prepared colloidal solution of COF particles in ACN (25 mg mL^–1^) with different sizes was dropped onto clean glass slides and dried in an oven at 120 °C for 5 min.

### Functionalizing of COF Particles with CTAB

COF particles were first washed three times with propylene carbonate (PCb) and subsequently redispersed in 10 mL of PCb at a concentration of 25 mg mL^–1^. For surface functionalization, 1 mL of a CTAB solution in PCb ([CTAB] = 2 mM) was added to each COF dispersion. The resulting mixtures were stirred for 1 h to allow adsorption of CTAB onto the particle surfaces. Finally, the particles were washed once more with PCb and redispersed in fresh PCb (25 mg mL^–1^).

### Electrophoretic Assembly of COF Particles

ITO-coated glass slides (for both line-patterned and unpatterned ones) were cleaned with isopropyl alcohol and Milli-Q water for 10 min. For electrophoretic deposition of the CTAB-functionalized COF particles in PCb (COF/PCb), 20 μL of as-prepared dispersions (25 mg mL^–1^) was injected between two ITO glasses that were separated from each other via spacers (120 μm thick). The two electrodes were connected with silver wires (0.25 mm diameter) to a stable DC voltage supply from a voltage amplifier (Krohn-Hite 7602M) controlled by a wave function generator (RS Pro RSDG 2042X). For the electrophoretic deposition of the particles, a maximum of ±2.0 V voltage was applied.

### Instrumentation

For optical measurements, a spectrometer (SR-4XR250-25, Ocean Optics Co.) and light generated by a tungsten–halogen light source (4.5 mW, HL-2000-LL-FHSA, Ocean Optics Co.) transmitted through a collimated optical fiber and collimated (λ = 360–2400 nm) were used. BF and DF optical microscope images were captured on a Zeiss Axio Imager.M2m microscope with a 10× objective connected to a Zeiss 305 color Axiocam and processed using ZEN microscopy software (version 3.9.101.02000). Scattering intensities of COF samples were collected using the Ocean Optics spectrometer integrated into the microscope under DF illumination.^54^ PXRD measurements were carried out on an Empyrean Panalytical in a reflection–transmission spinner configuration. The anode material is Cu, step size [°2θ] is 0.0130, generator settings were 40 mA and 45 kV, and the measurement temperature [°C] was 25. FE-SEM (Zeiss Supra 55 VP) operating at an acceleration voltage of 5 kV was applied to characterize the morphology and size of the crystals. Before each characterization procedure, 20 μL of COF particle samples (25 mg mL^–1^) with varying sizes in ACN was deposited onto FE-SEM grids and left to dry at room temperature. DLS studies were carried out with a Malvern Zetasizer Nano ZS. To determine the hydrodynamic diameter and zeta potential of the particles of varying sizes, 800 μL of COF particle samples (25 mg mL^–1^) with varying sizes in ACN was transferred into disposable cuvettes (DTS1070), and the characterization was performed at room temperature in triplicate. Raman spectra of the particles were collected in WITec alpha 300A Raman/AFM coupled with a CCD detector and 300 g mm^–1^ grating (785 nm, 10 mW, 10 s integration time, 3 accumulations).

## Supplementary Material



## Notes

Scanning electron micrographs, optical micrographs and additional photographs, reflectance spectra, CIE-color chromaticity plots, Raman spectrum, and N_2_ sorption isotherms (PDF)
