# Supplementary material for: Hidden Structural Colors from Bistable, Electrically Driven Covalent Organic Framework Photonic Assemblies for Secure Optical Encoding
Source: ACS Nano. 2025 Dec 18;20(1):1555–65. doi: 10.1021/acsnano.5c18545 (PMC12810473; doi:10.1021/acsnano.5c18545)
Supplement: Supplementary file 1 [file nn5c18545_si_001.pdf]

Supporting Information for

Hidden Structural Colors from Bistable, Electrically Driven Covalent Organic Framework Photonic Assemblies for Secure Optical Encoding

Tolga Zorlu[†], *Flora Schöfbeck*[†,§], *Julian Lemmel*[≠], *Daoming Sun*[‡], *Tanja Eder*[†], *Michael R. Reithofer*[‡,*] *and Jia Min Chin*[†, ¥,*]

† Department of Functional Materials and Catalysis, University of Vienna, 1090 Vienna, Austria

§ Vienna Doctoral School in Chemistry (DoSChem), University of Vienna, 1090 Vienna, Austria

‡ Institute of Inorganic Chemistry, Faculty of Chemistry, University of Vienna, 1090 Vienna, Austria

≠ Cyber-Physical-Systems-Group, Faculty of Informatics, Technical University Vienna, 1040 Vienna, Austria

¥ Wolfgang Pauli Institute, 1090 Vienna, Austria

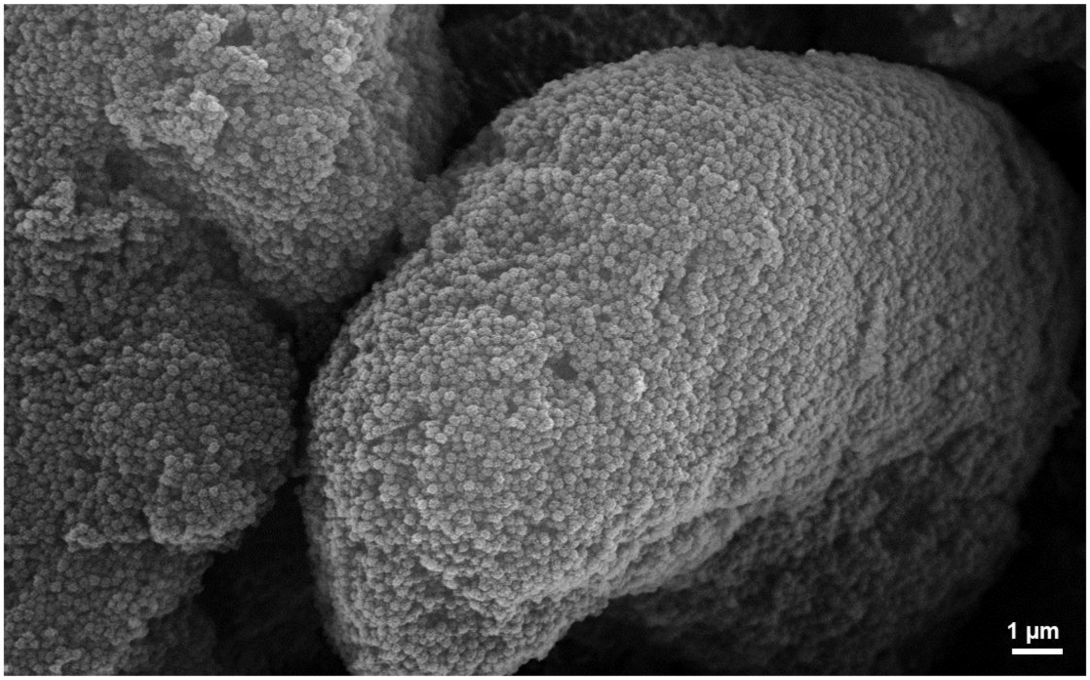

Figure S1. FE-SEM images of synthesized COF particles (187 ± 13 nm) (200 µL PVP).

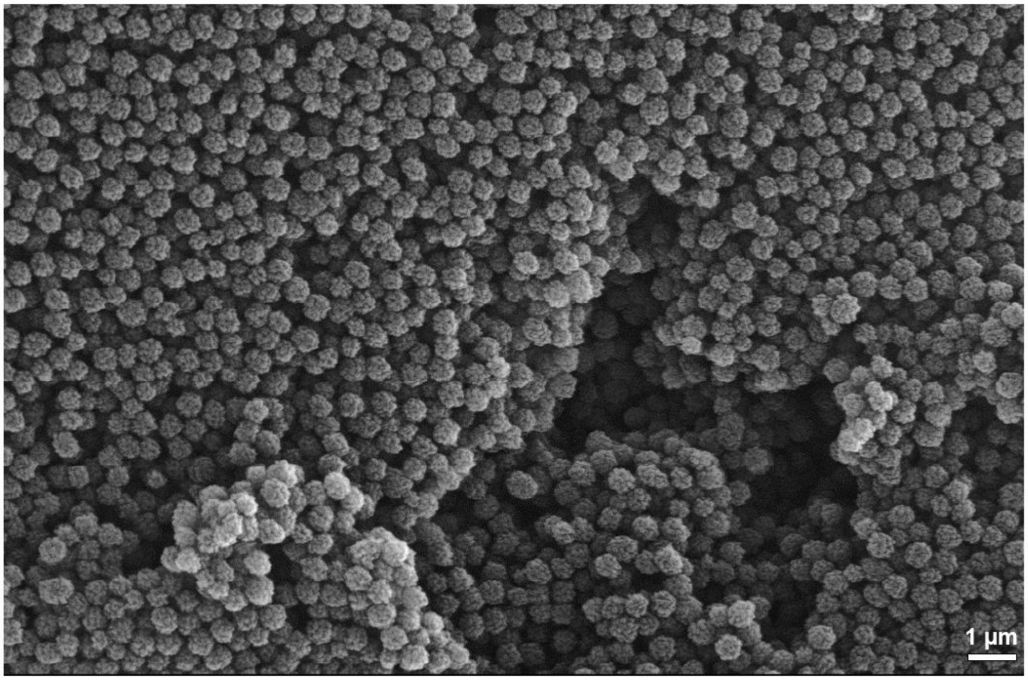

Figure S2. FE-SEM images of synthesized COF particles (212 ± 8 nm) (100 μL PVP).

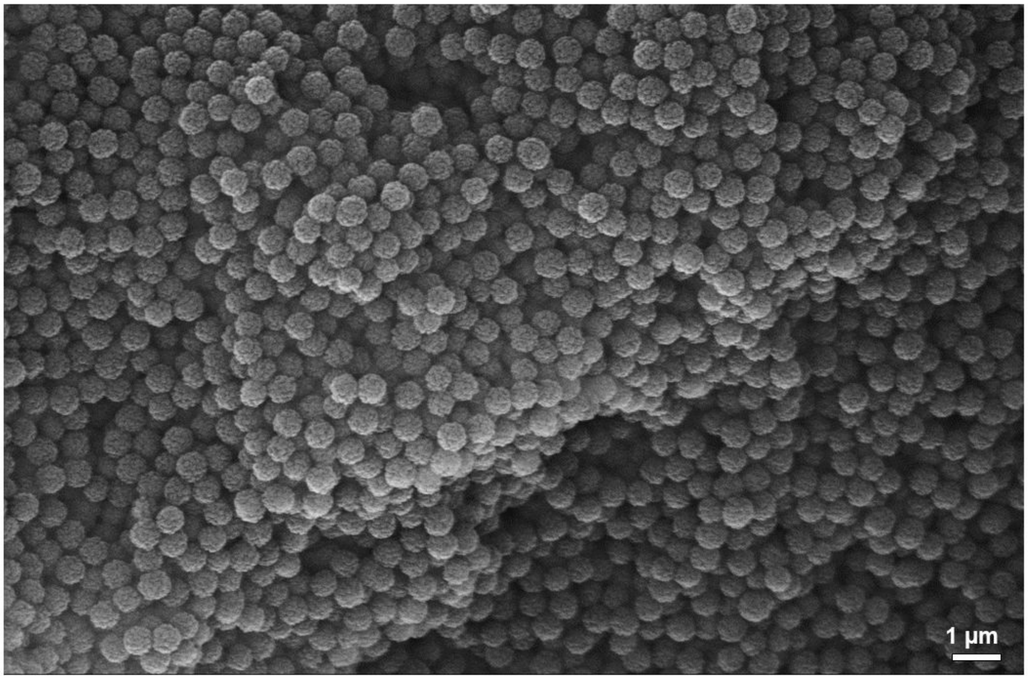

Figure S3. FE-SEM images of synthesized COF particles (257 ± 8 nm) (0 μL PVP).

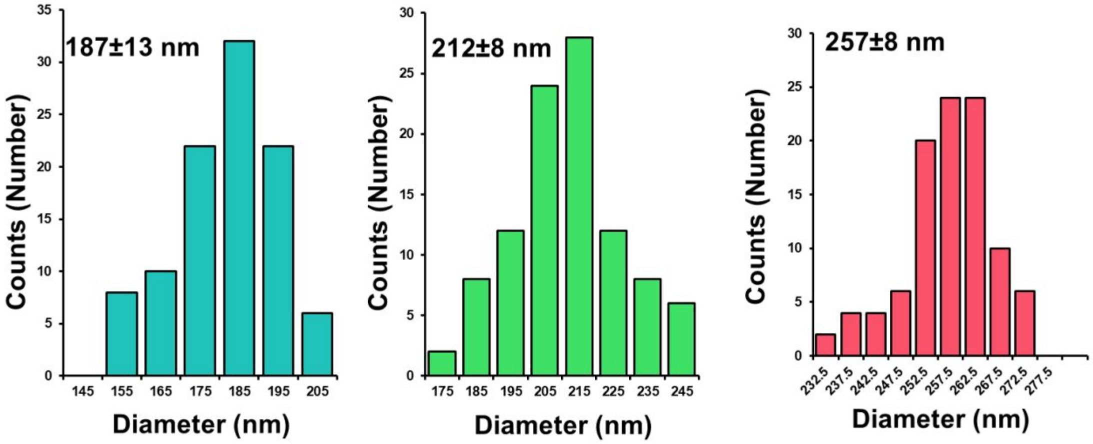

Figure S4. Corresponding histograms of as-prepared COF particles.

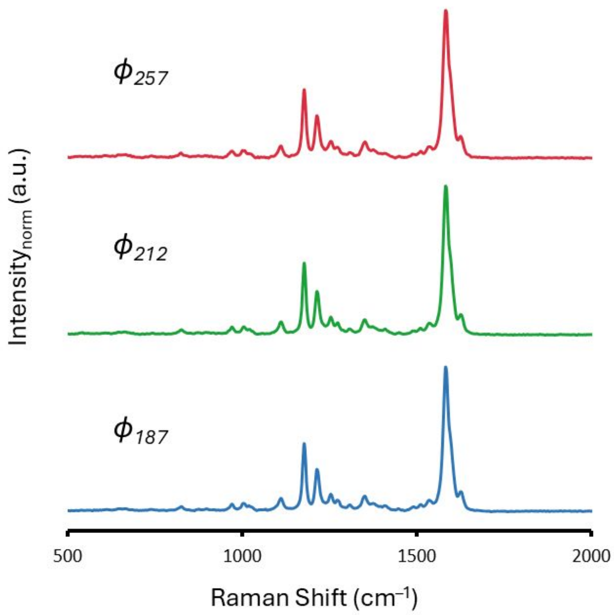

Figure S5. Raman spectra of COF particles.

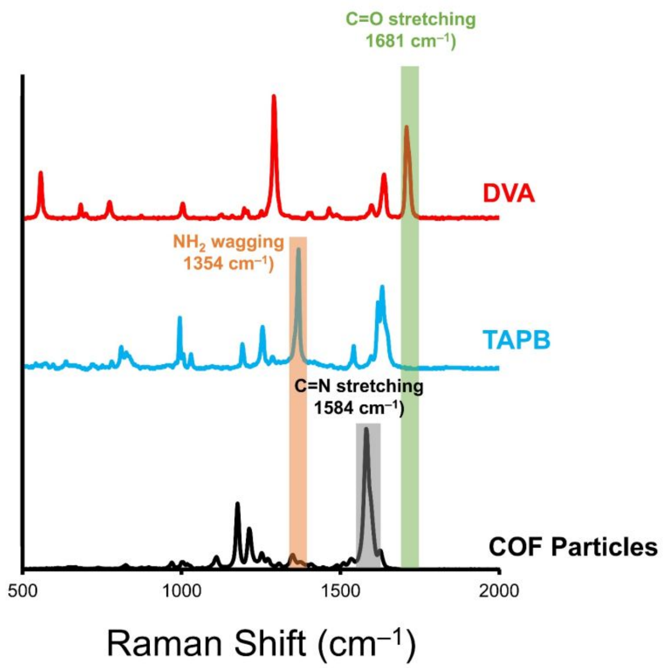

Figure S6. Raman spectra of both monomers and as-prepared COF particles (ϕ_{257}).

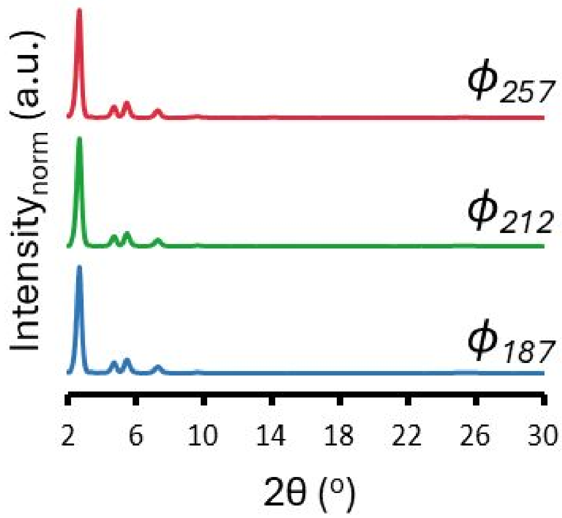

Figure S7. PXRD pattern of as-prepared COF particles.

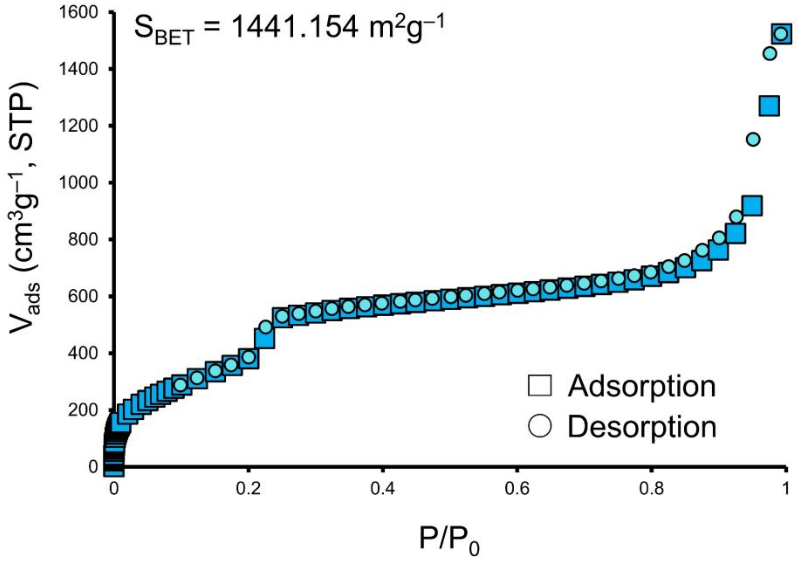

Figure S8. N_2 sorption isotherms @77 K of as-prepared COF particles (ϕ_{187}) Squares and circles represent adsorption and desorption, respectively. BET surface areas (S_{BET}) of the particles are also given inset.

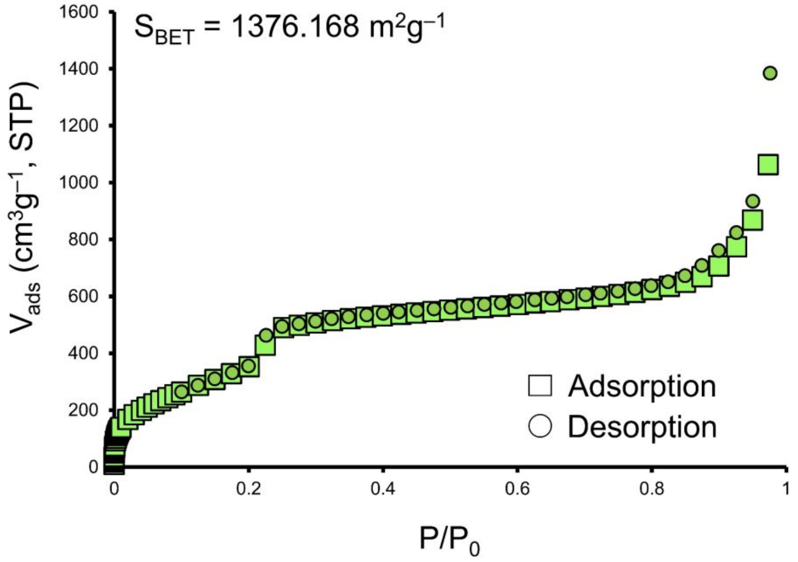

Figure S9. N_2 sorption isotherms @77 K of as-prepared COF particles (ϕ_{212}) Squares and circles represent adsorption and desorption, respectively. BET surface areas (S_{BET}) of the particles are also given inset.

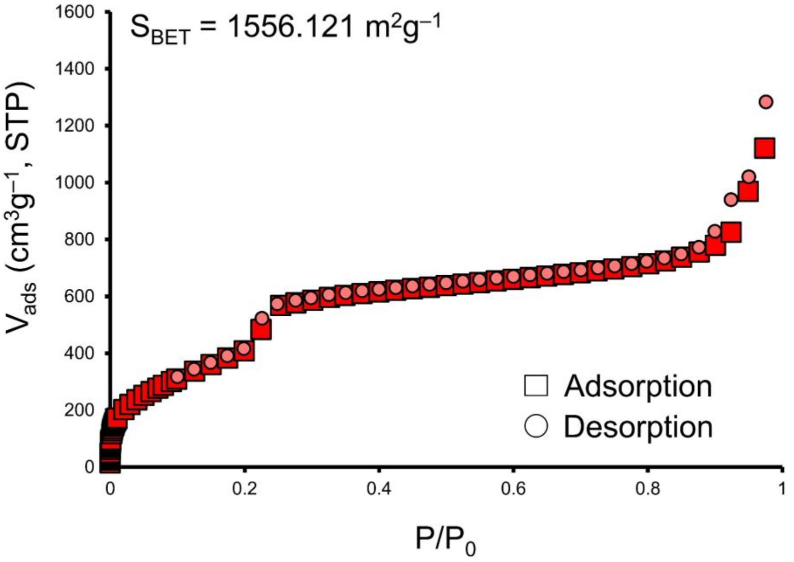

Figure S10. N_2 sorption isotherms @77 K of as-prepared COF particles (ϕ_{257}) Squares and circles represent adsorption and desorption, respectively. BET surface areas (S_{BET}) of the particles are also given inset.

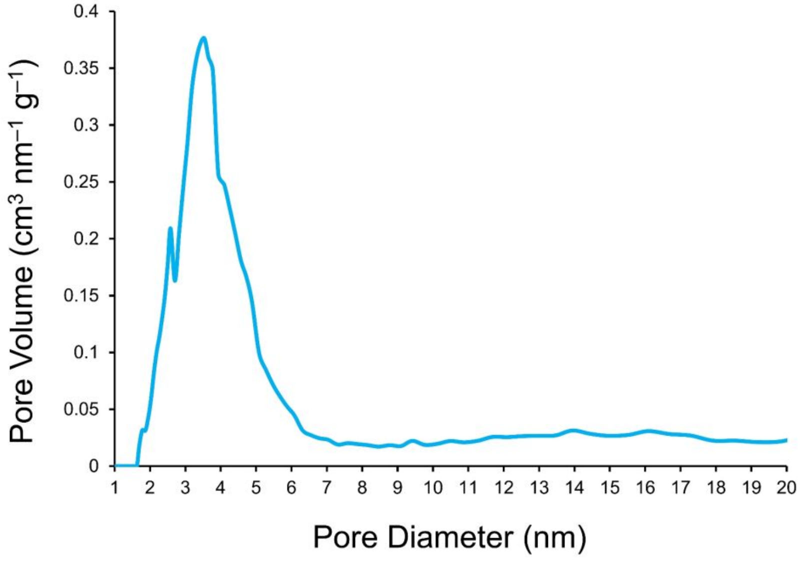

Figure S11. DFT pore size analysis of COF particles (ϕ_{187}).

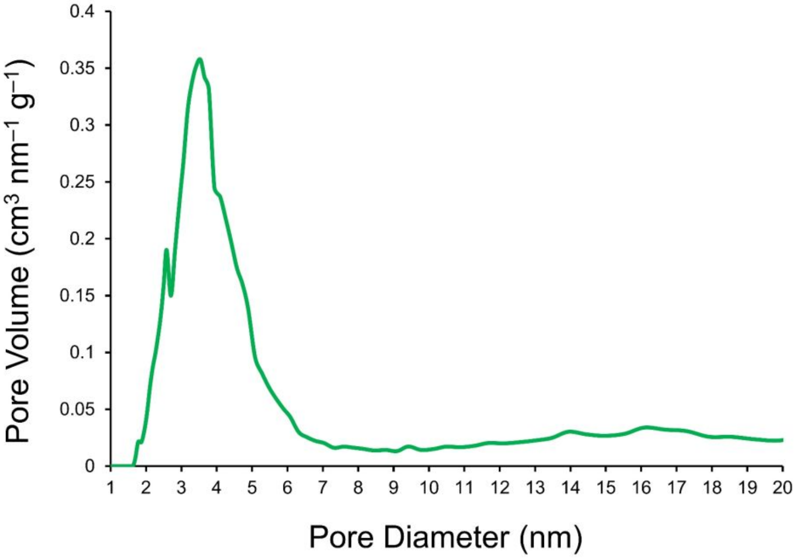

Figure S12. DFT pore size analysis of COF particles (ϕ_{212}).

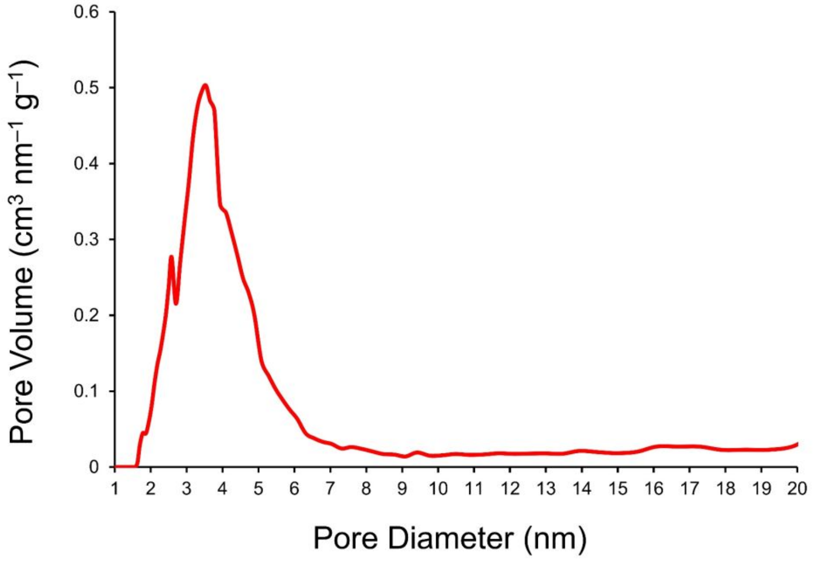

Figure S13. DFT pore size analysis of COF particles (ϕ_{257}).

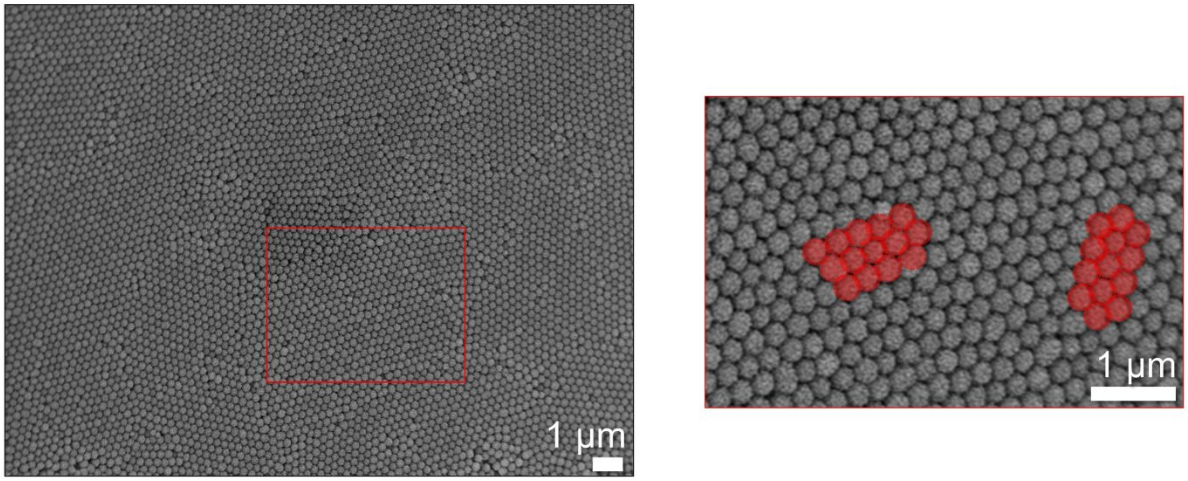

Figure S14. (left) FE-SEM images of a self-assembled superstructure comprising COF particles (ϕ_{257}). A higher magnified section (right) shows the (*111*) planes of COF particles (red marks on the image have been digitally added to better visualize the (*111*) planes).

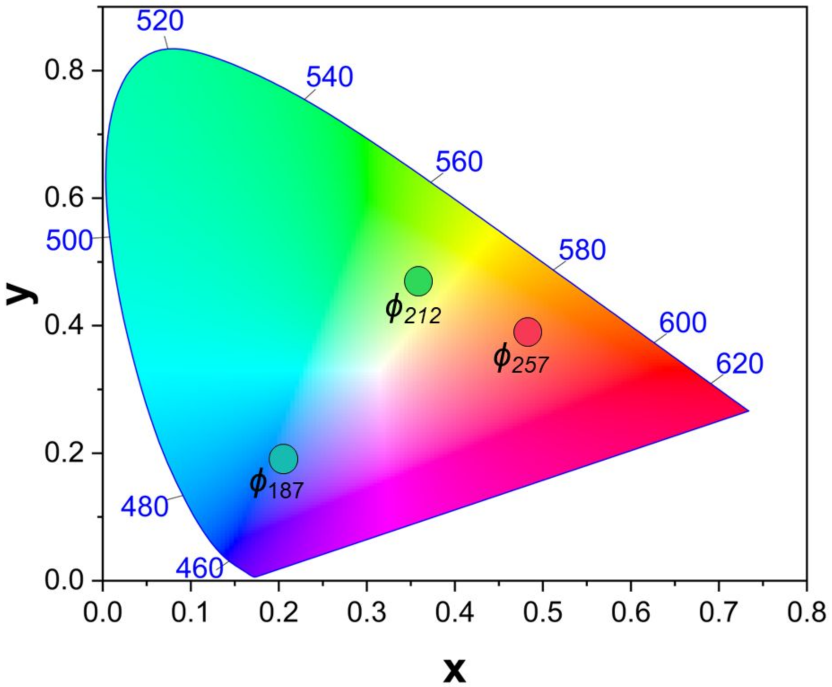

Figure S15. CIE-1931 chromaticity diagram based on the normalized reflectance spectra at θ = 0° of the as-prepared dry photonic COF superstructures with different particle sizes.

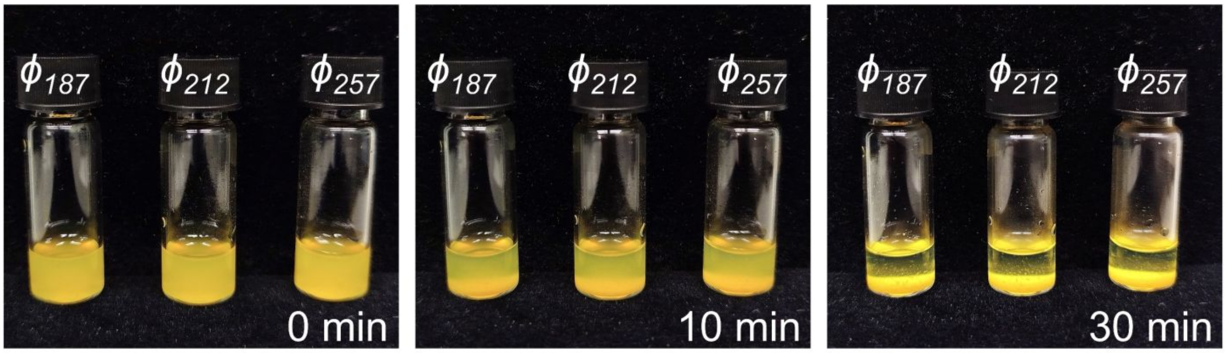

Figure S16. Colloidal stabilities of COF particles in PCb without post-synthetic modification.

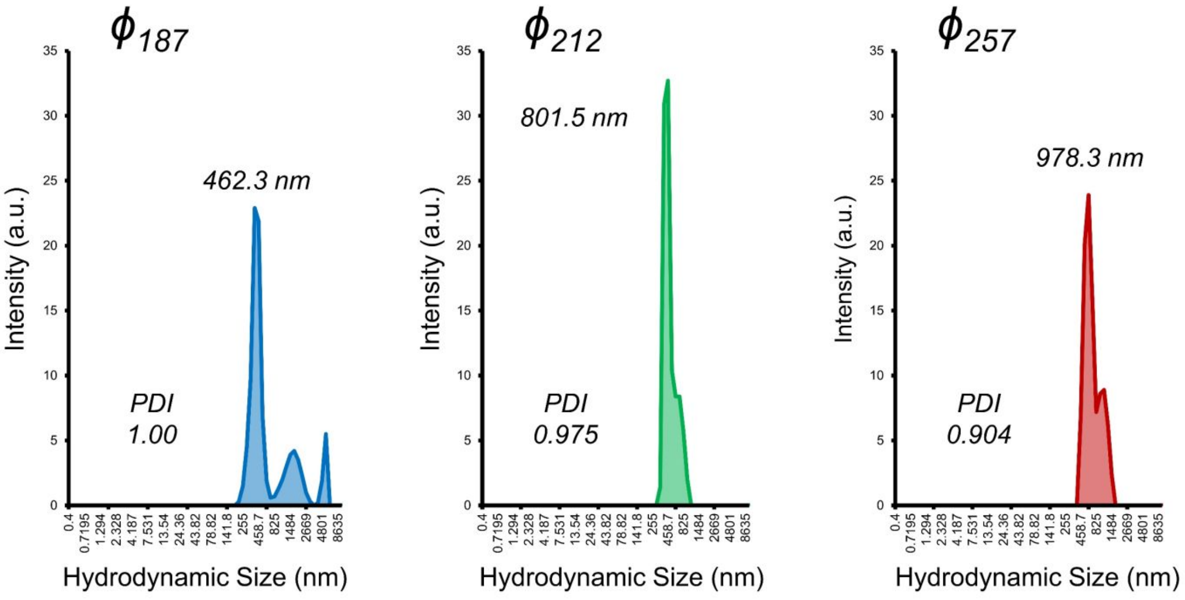

Figure S17. Hydrodynamic sizes of COF particles in PCb without post-synthetic modification. Polydispersity index (PDI) values of the particles are also given inset.

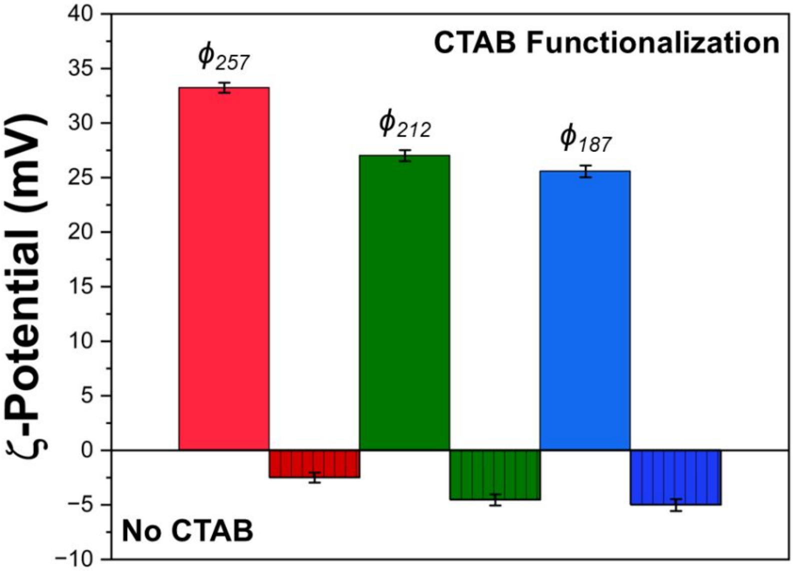

Figure S18. Zeta potential values of functionalized and non-functionalized COF particles.

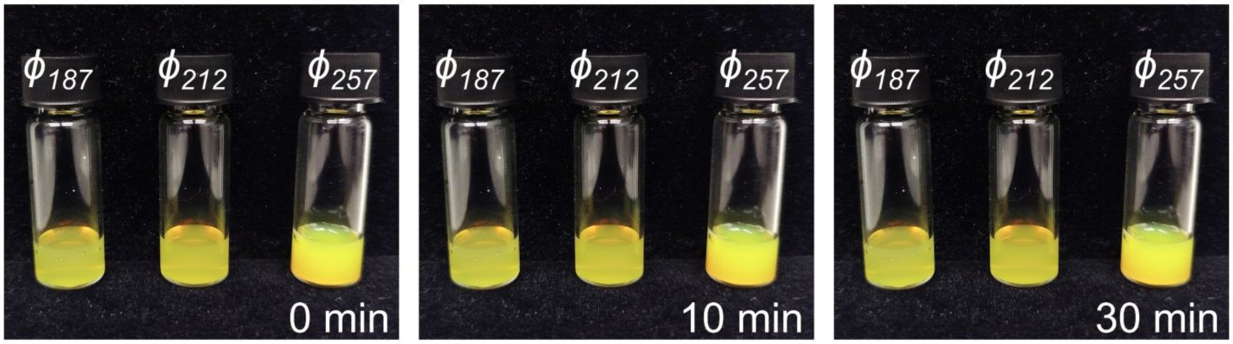

Figure S19. Colloidal stabilities of COF particles in PCb after CTAB treatment. $[CTAB]_{final}$ = 5 mM).

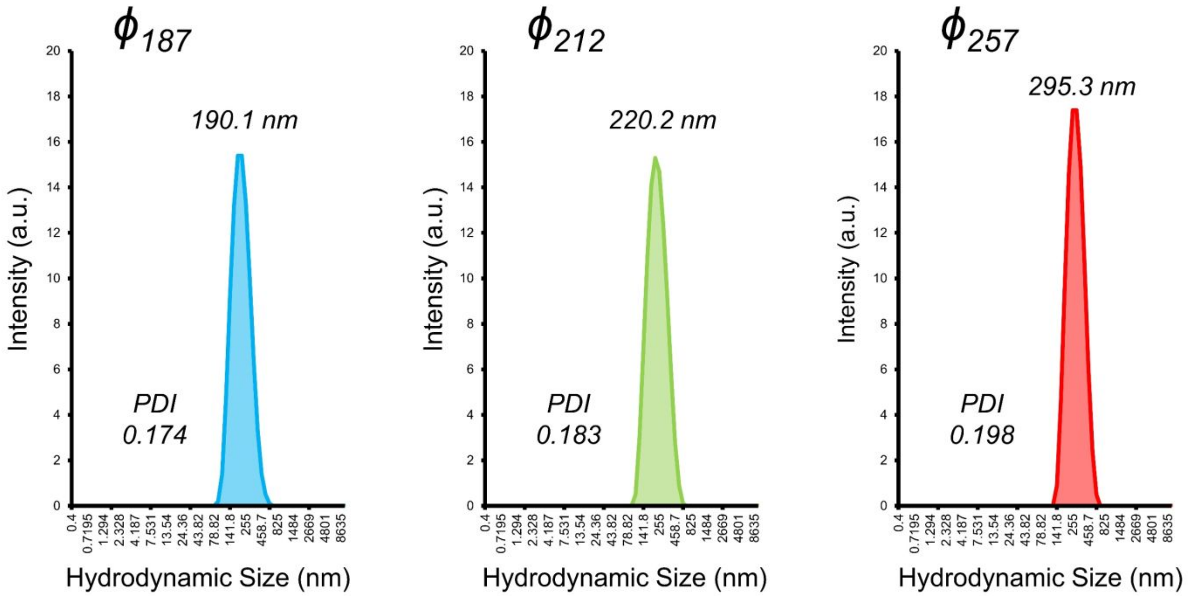

Figure S20. Hydrodynamic sizes of COF particles in PCb after CTAB treatment. $[CTAB]_{final}$ = 5 mM. PDI values of the particles are also given inset.

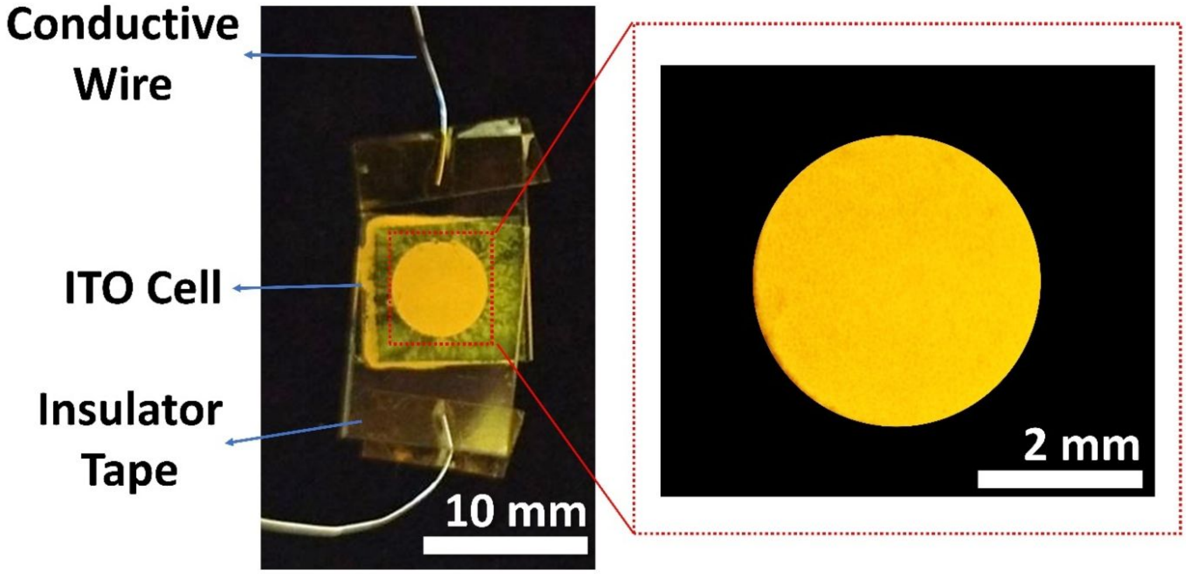

Figure S21. A photograph of an electrophoretic cell comprising two ITO-coated glass substrates, a spacer, two conductive wires, and a COF/PCb dispersion, shown before and after the application of a DC electric field. The region marked with a red dotted outline has been magnified to highlight the COF/PCb dispersion within the cell. The surrounding black background has been digitally added for visual clarity.

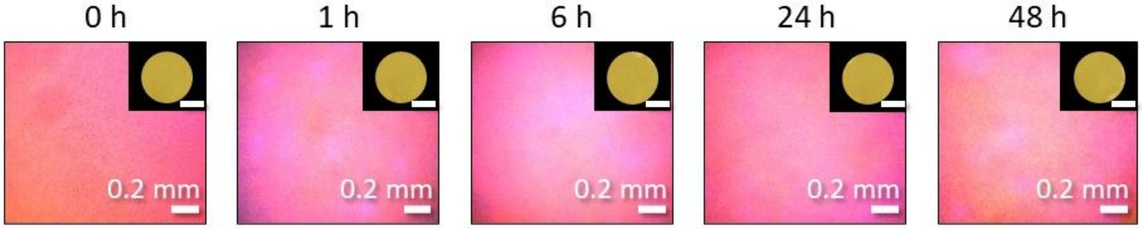

Figure S22. Time-dependent BF optical micrographs and macrographs (inset) showing the structural color stability in the COF/PCb dispersion (ϕ_{257}) at $\theta = 0°$ after the EF is switched off.

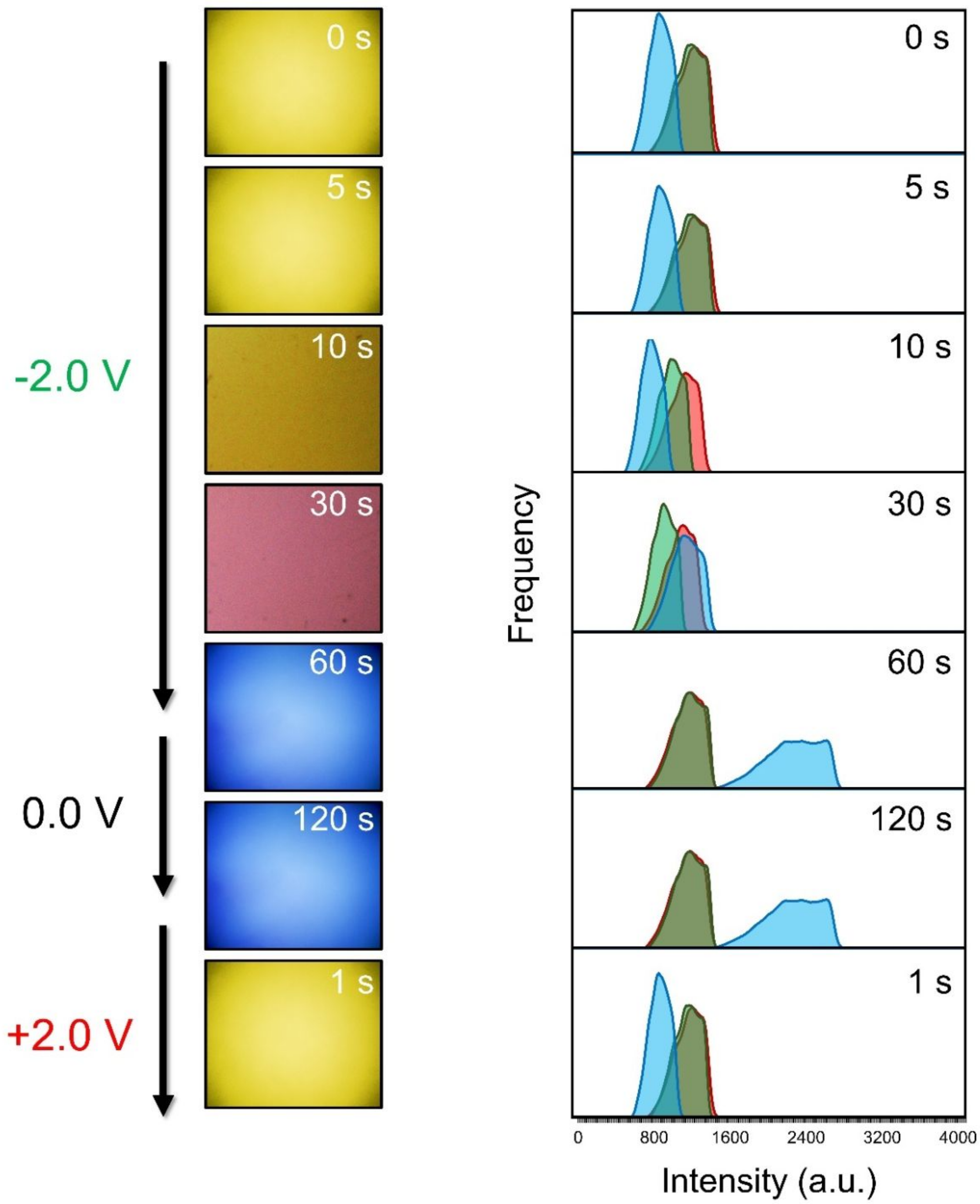

Figure S23. Time-dependent BF optical microscope images of evolution of structural color changes in the COF/PCb (ϕ_{187}) at $\theta = 0°$ with corresponding RGB histogram

.

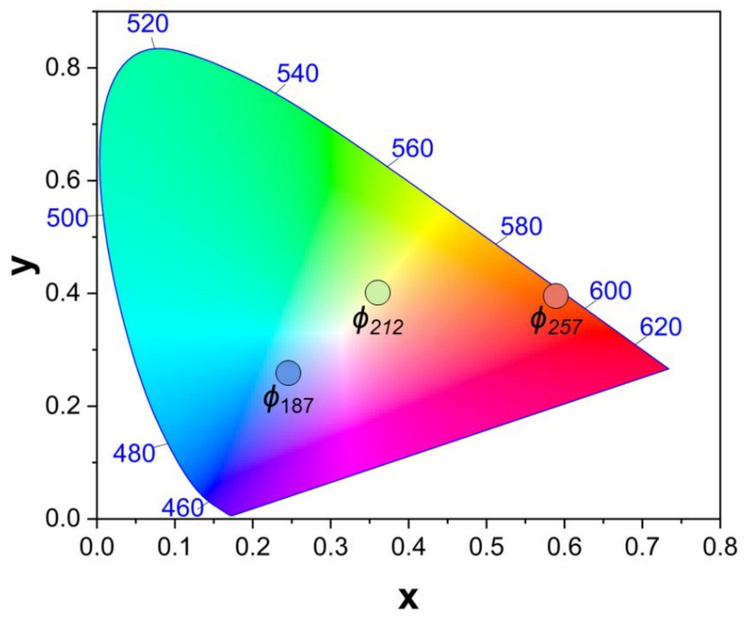

Figure S24. CIE-1931 chromaticity diagram, derived from the normalized reflectance spectra, for COF/PCb of different particle sizes after EF application.

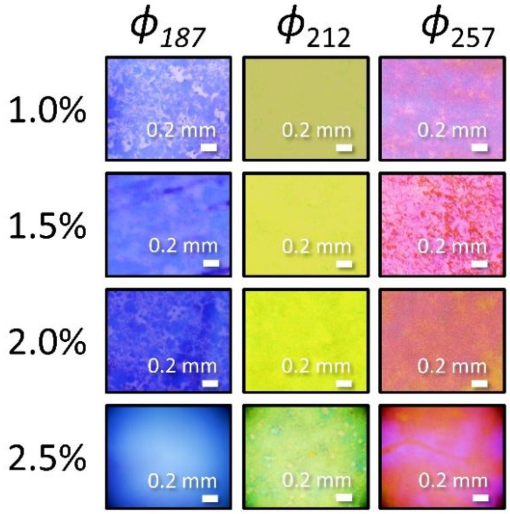

Figure S25. BF microscope images of evolution of structural color changes of different weight percentages of COF/PCb (ϕ_{187}, ϕ_{212}, and ϕ_{257}) at $\theta = 0°$.

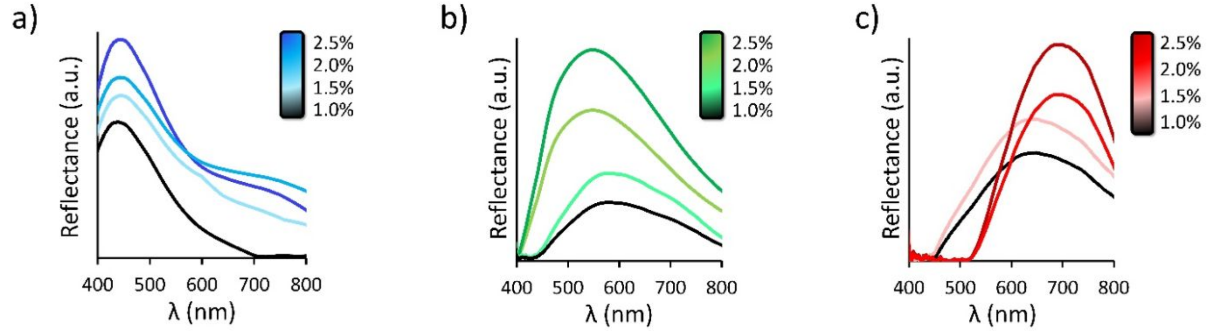

Figure S26. Corresponding reflectance spectra of COF/PCb with different weight percentages at $\theta = 0°$: a) ϕ_{187}, b) ϕ_{212}, and c) ϕ_{257}, respectively).

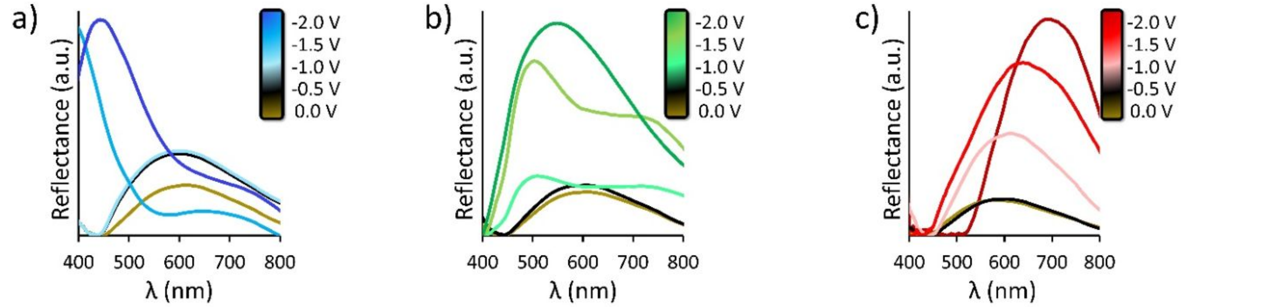

Figure S27. Corresponding reflectance spectra of COF/PCb with different voltages at θ = 0°: a) ϕ_{187}, b) ϕ_{212}, and c) ϕ_{257}, respectively).

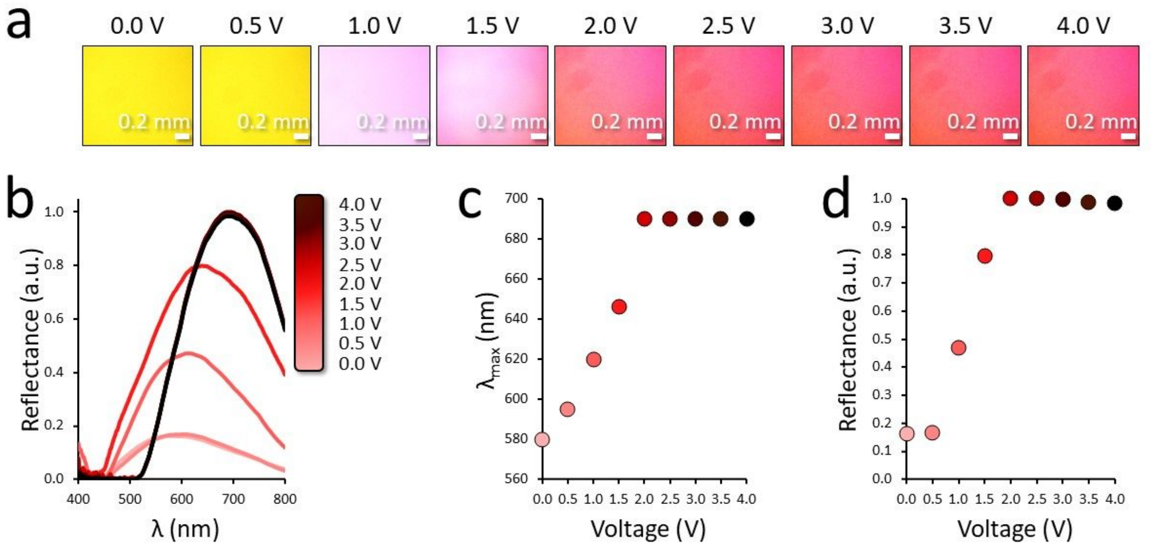

Figure S28. a) BF optical micrographs of voltage-dependent colorization of the COF/PCb dispersion (ϕ_{257}) in the electrophoretic cell. b) Corresponding reflectance spectra of COF/PCb at different voltages at $\theta = 0°$. c) Plot showing the change in λ_{max} as a function of voltage. (d) Plot showing the change in reflectance intensity as a function of voltage.

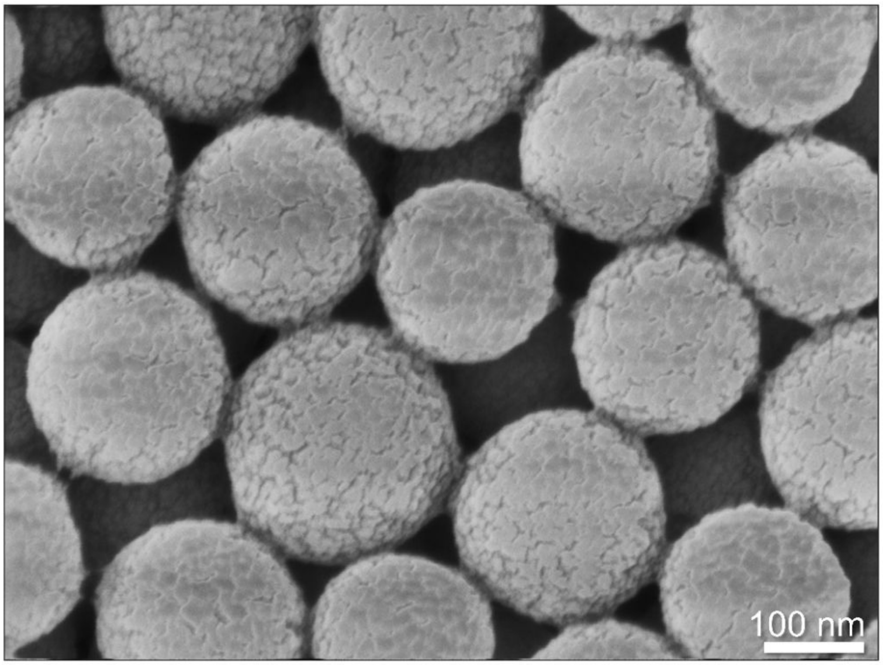

Figure S29. FE-SEM images of synthesized COF particles (5 minutes).

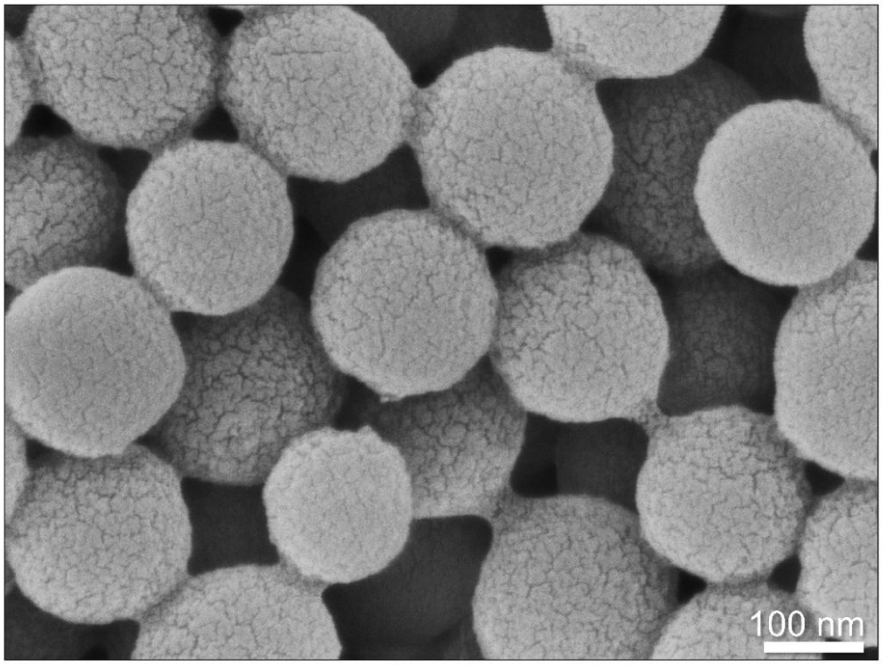

Figure S30. FE-SEM images of synthesized COF particles (30 minutes).

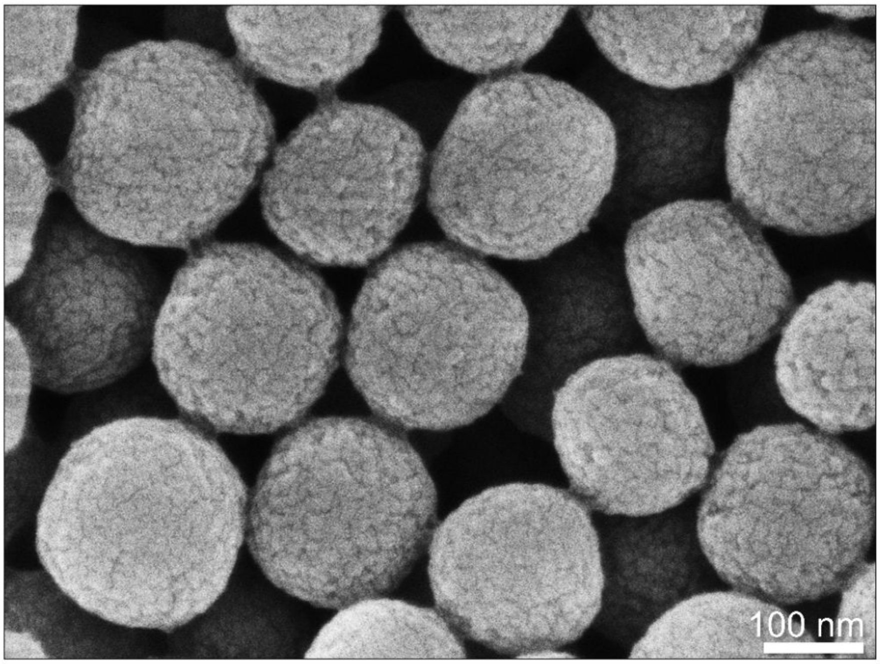

Figure S31. FE-SEM images of synthesized COF particles (60 minutes).

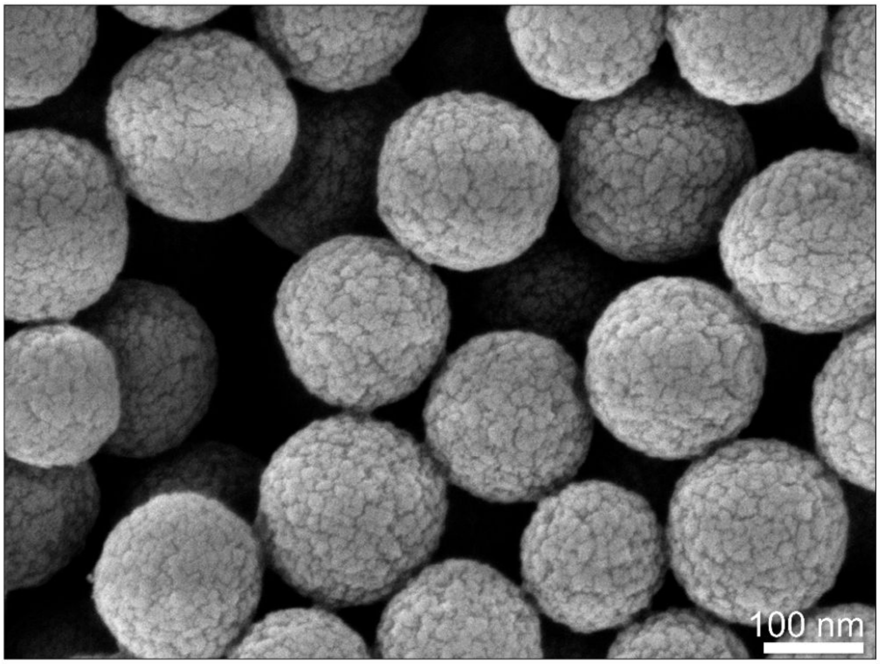

Figure S32. FE-SEM images of synthesized COF particles (360 minutes).

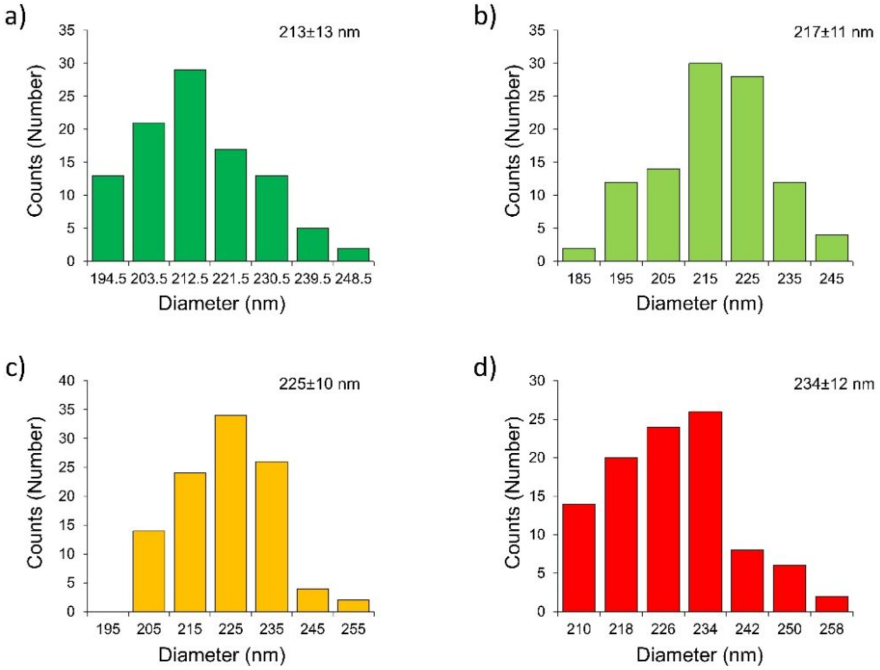

Figure S33. Size histograms of as-synthesized COF particles at different times: a) 5, b) 30, c) 60, and d) 360 minutes.

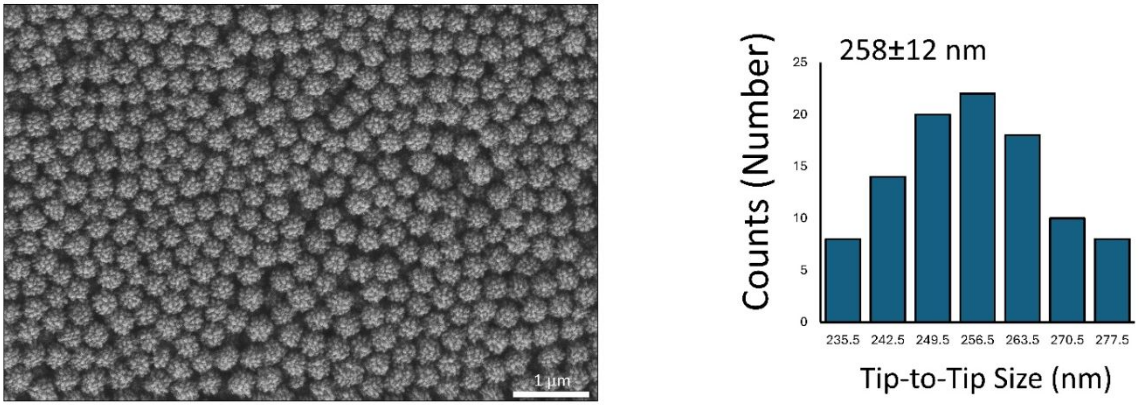

Figure S34. FE-SEM images of synthesized COF particles (72 hours) (PVP = 0 mg L^{-1}) and corresponding histograms.

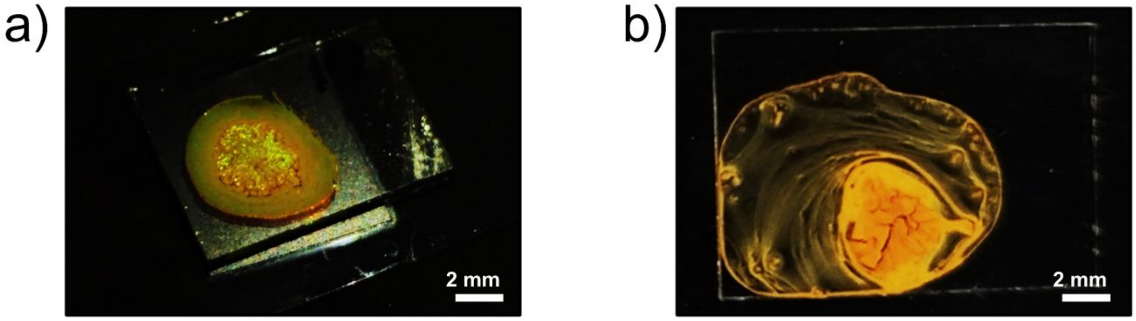

Figure S35. BF micrographs of a) dried ϕ_{213} (smooth) and b) ϕ_{212} (rough) COF superstructures.

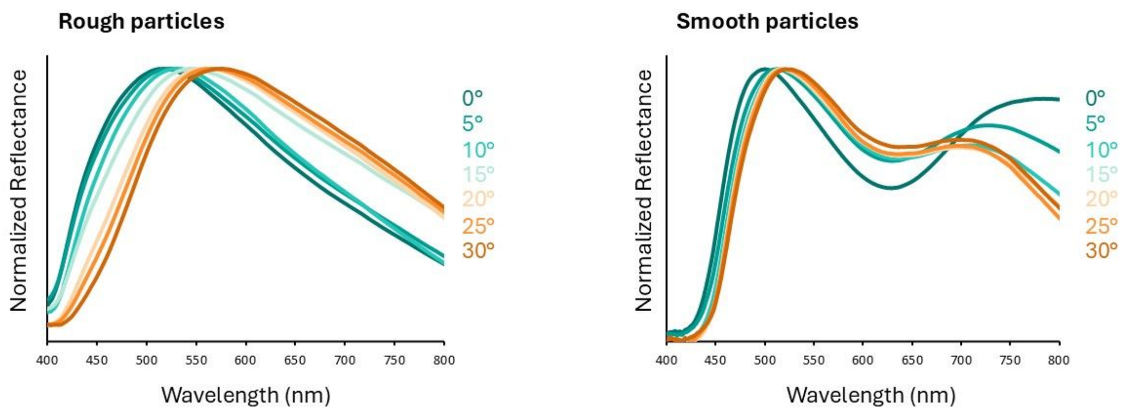

Figure S36. Angle-resolved reflectance spectra of rough (212 ± 8) and smooth (213 ± 13) COF particles at varying incidence angles.

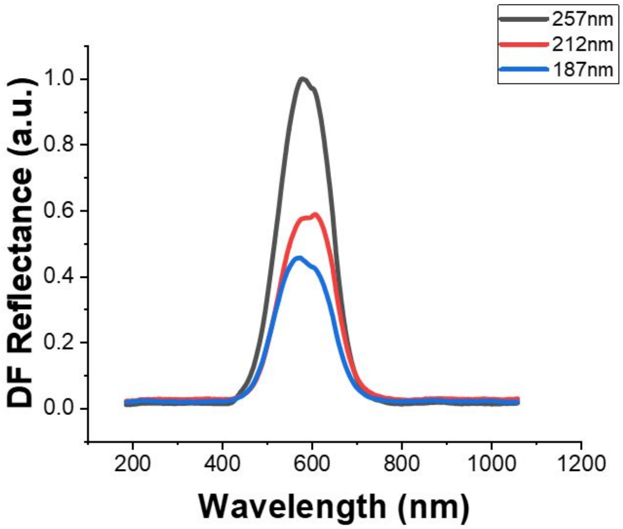

Figure S37. DF scattering spectrum of COF particles with different sizes.

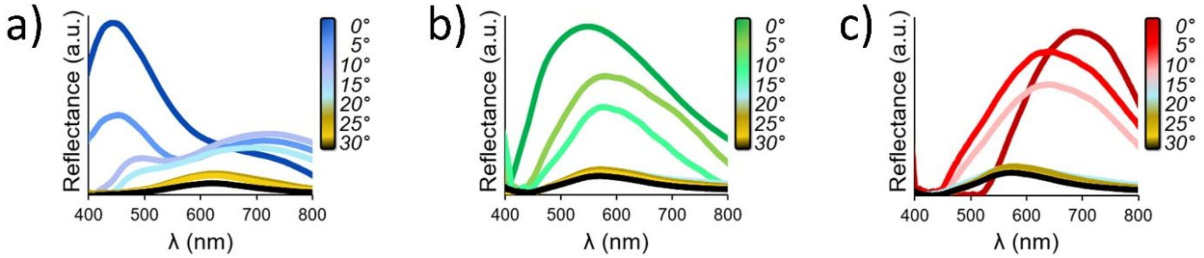

Figure S38. Reflectance spectra of COF/PCb at varying incidence angles: a) ϕ_{187}, b) ϕ_{212}, and c) ϕ_{257}.

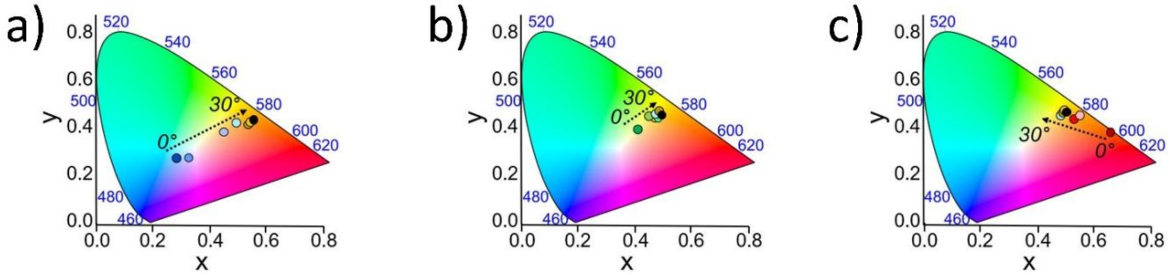

Figure S39. CIE-1931 diagrams COF/PCb under EF at varying incidence angles. a) ϕ_{187}, b) ϕ_{212}, and c) ϕ_{257}.

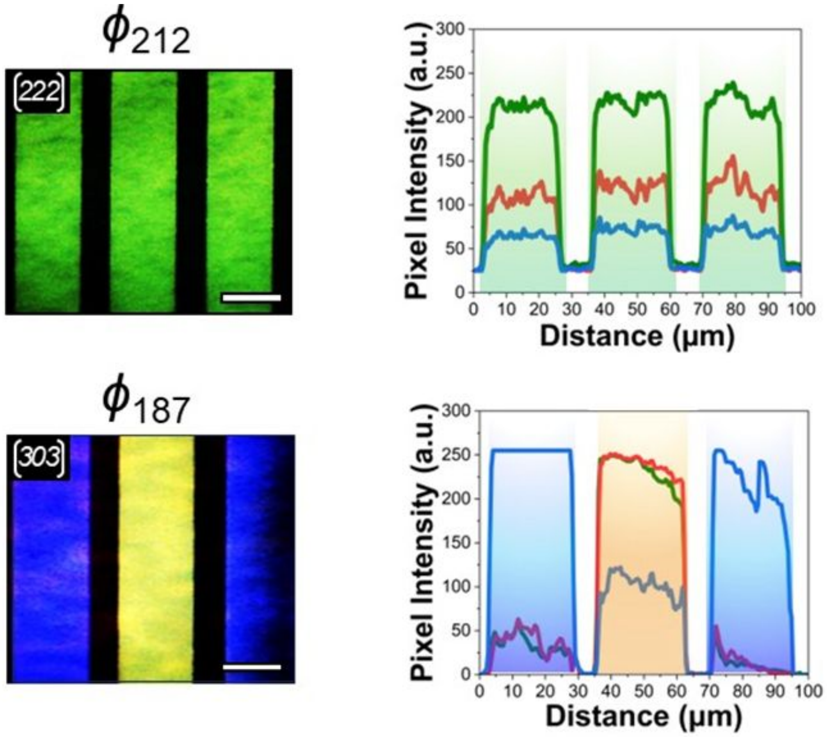

Figure S40. BF patterned images and pixel intensity–distance plots of ϕ_{187} and ϕ_{212} samples.

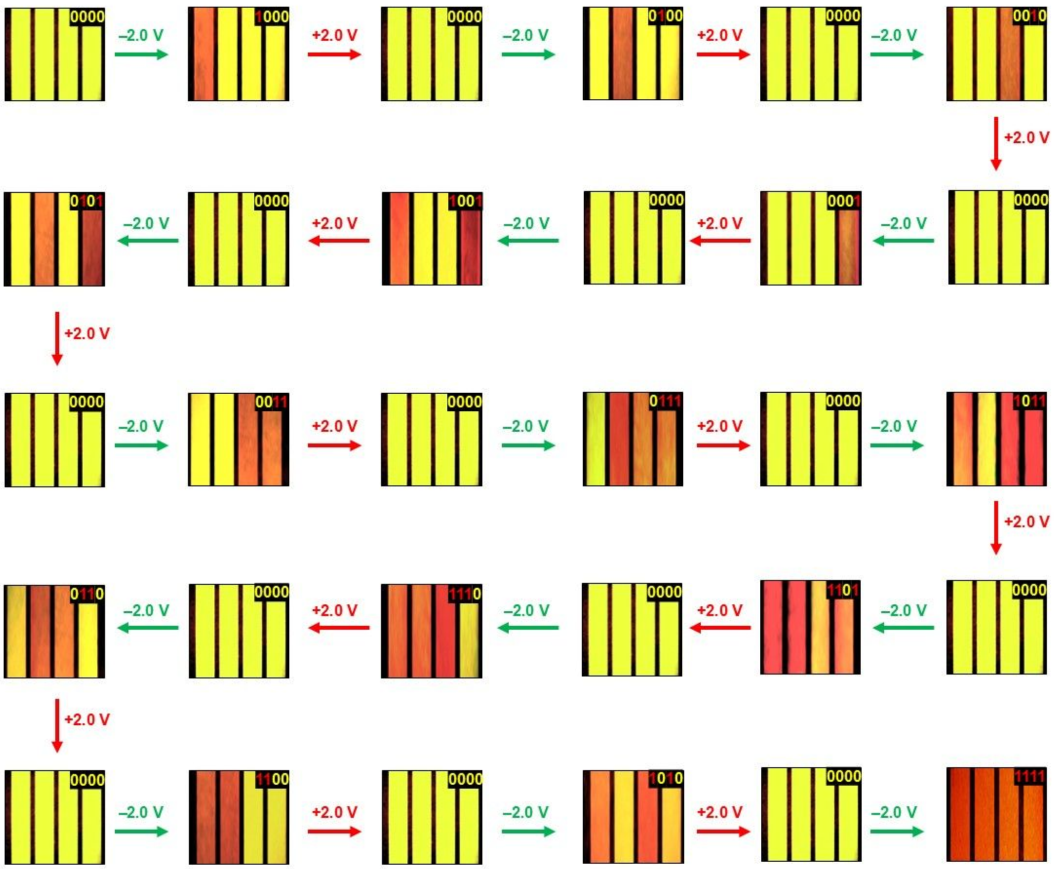

Figure S41. BF images obtained using ϕ_{257}, showing all possible combinations in accordance with the write/erase/rewrite cycle.

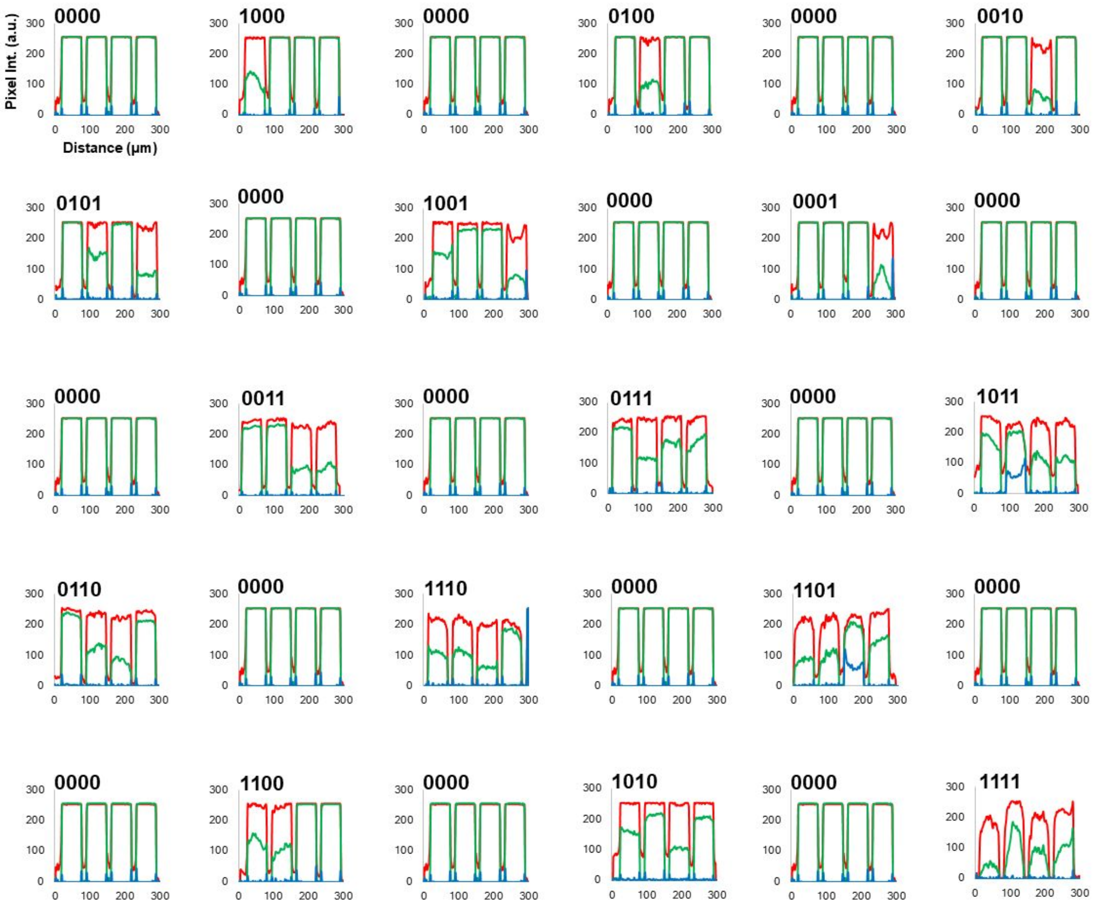

Figure S42. Pixel intensity–distance plots obtained using ϕ_{257}, showing all possible combinations in accordance with the write/erase/rewrite cycle.

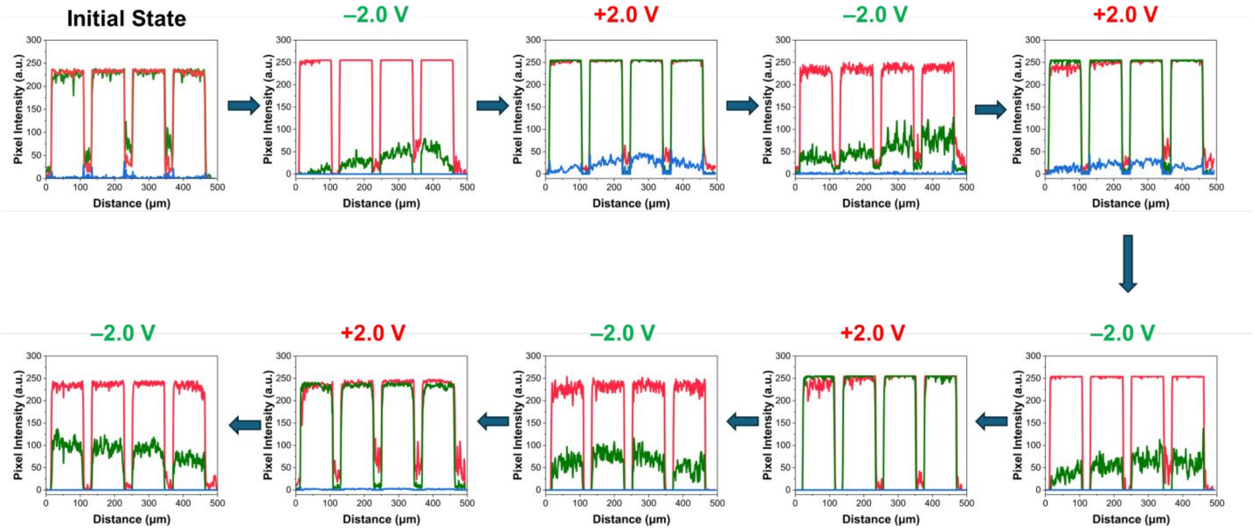

Figure S43. Pixel intensity–distance plots obtained using ϕ_{257} after different the write/erase/rewrite cycle.

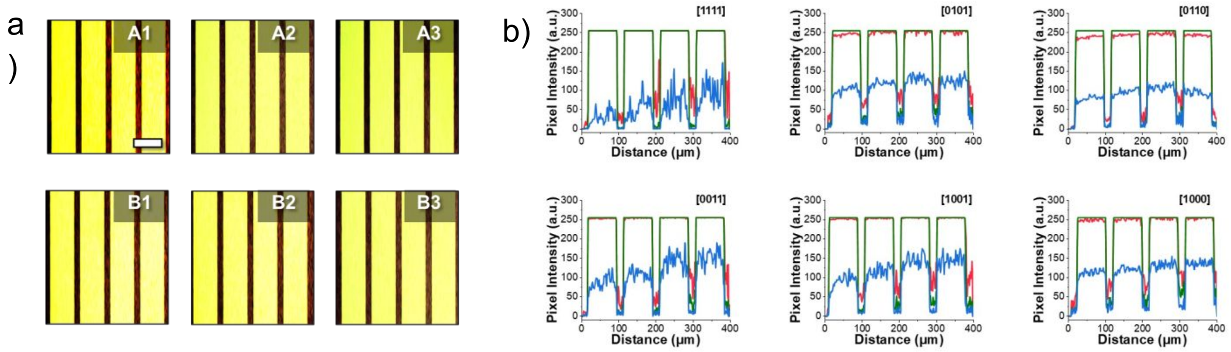

Figure S44. a) BF optical micrographs of ϕ_{257} superstructures corresponding to the erased state, or [0000 0000 0000 0000 0000 0000]. b) Pixel intensity–distance plots obtained using ϕ_{257} corresponding to the micrographs in a).
